# Relationships between species richness and biomass production are context dependent in grasslands differing in land-use and seed addition

**DOI:** 10.1038/s41598-023-47020-z

**Published:** 2023-11-11

**Authors:** Karl Andraczek, Alexandra Weigelt, Cristóbal J. Bottero Cantuarias, Markus Fischer, Judith Hinderling, Daniel Prati, Esther M. N. Rauwolf, Fons van der Plas

**Affiliations:** 1 Faculty of Life Sciences, Systematic Botany and Functional Biodiversity, Leipzig University, Johannisallee 21, 04103 Leipzig, Germany; 2 German Centre for Integrative Biodiversity Research (iDiv) Halle-Jena-Leipzig, Puschstr. 4, 03401 Leipzig, Germany; 3 Institute of Plant Sciences, University of Bern, Hochschulstrasse 4, 3012 Bern, Switzerland; 4 Plant Ecology and Nature Conservation Group, Wageningen University, P.O. Box 47, Wageningen, The Netherlands

**Keywords:** Biodiversity, Community ecology, Ecosystem ecology

## Abstract

Despite evidence from grasslands experiments suggesting that plant species loss reduces biomass production, the strength of biodiversity-ecosystem functioning relationships in managed grasslands is still debated. High land-use intensity and reduced species pools are often suggested to make relationships between biodiversity and productivity less positive or even negative, but concrete evidence is still scarce. We investigated biodiversity-productivity relationships over two years in 150 managed grasslands in Germany. Specifically, we distinguished between relationships of biodiversity and biomass production in managed grasslands (1) varying in land-use intensity (e.g. of mowing, grazing and/or fertilization), (2) where land-use intensity is experimentally reduced, and (3) where additionally to land-use reductions, species pools are enlarged by seed addition. Among grasslands varying in land-use intensity, we found negative biodiversity-productivity relationships. Land-use reduction weakened these relationships, towards neutral, and sometimes, even positive relationships. Seed addition reduced species pool limitations, but this did not strengthen biodiversity-productivity relationships. Our findings indicate that land-use intensity is an important factor explaining the predominantly negative biodiversity-productivity relationships in managed grasslands. While we did not find that species pool limitations weakened biodiversity-productivity relationships, our results are based on a two-year-old experiment, possibly such effects are only visible in the long-term. Ultimately, advancing insights on biodiversity-ecosystem functioning relationships helps us to understand under which conditions agricultural production may benefit from promoting biodiversity.

Global biodiversity is declining at an unprecedented rate due to various human activities^1,2^. Evidence from experimental grasslands shows that high plant biodiversity often has positive effects on multiple aspects of ecosystem functioning, including biomass production^3–7^. Thus, a concern is that ecosystem functioning will be hampered by biodiversity loss^5^. However, most evidence on positive relationships between plant species richness and biomass production is based on field experiments, where random biodiversity loss is simulated and spatial variation in abiotic conditions and management is strongly reduced in comparison to managed grasslands. Hence, there has been controversy on whether these biodiversity effects on biomass production are strong enough to be relevant in managed grasslands^7–9^, being the predominant grassland type in Europe^10^ and where management incentives focus on a few, fast growing plant species that maximise biomass production. In particular, the question is whether positive effects of species richness and biomass production can compensate for typically contrasting responses of biomass production (mostly positive) and plant species richness (mostly negative) to increasing grassland land-use intensity, such as increasing mowing, grazing or fertilization intensity^11,12^.

Aiming to unravel the highly variable biodiversity-productivity relationships found in previous observational studies, Schmid^12^ proposed a theoretical framework (Fig. 1) in which he argued that variability in relationships found in observational studies arises from variation in grassland land-use intensity^13^. Particularly mowing, grazing and fertilization intensity are the most important components of land-use in central European grasslands^14^, influencing both plant species richness and biomass production^14–16^. In managed grasslands differing in site fertility or fertilizer application, relationships between plant species richness and biomass production are hypothesized to follow a ‘hump-backed’ pattern (mathematically meaning a concave down parabola, red line Fig. 1,^17^, but see^18^), as biomass production typically increase, while species richness decreases with fertilizer application and associated increased harvesting (e.g. mowing/ grazing) activities (Fig. 1, side panel 1). This decrease in species richness can result from increasing competitive dominance of one or a few species^19–21^, as only a few species can tolerate high disturbance intensities. Additionally, species richness has also been shown to be associated with decreased seed production or suppression of more palatable species^15^. Highly fertilized grasslands are typically also frequently mown and/or grazed in order to feed livestock. While mowing and grazing can oppose some of the negative effects of fertilizer application on plant biodiversity^22,23^, the overall, negative effects of fertilizer application (or nutrient input by grazing mammals) in intensively managed grasslands generally outweigh the more moderate, positive effects of mowing and grazing^15,16^.

**Figure 1:**
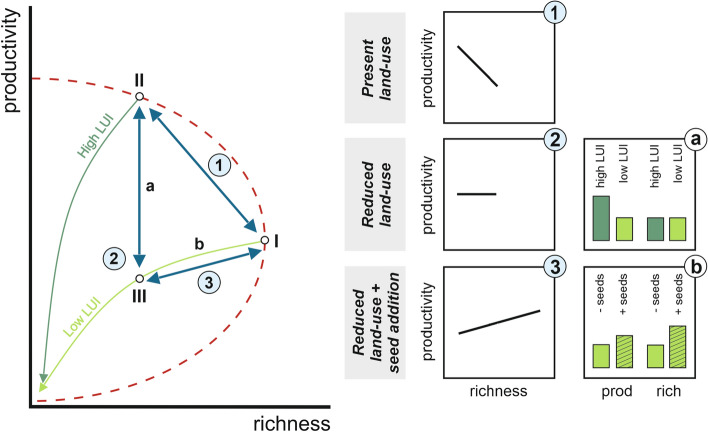
Conceptional framework showing the hypothesized relationships between plant species richness and productivity in managed grasslands (after^12^, 2002, modified). Main panel: If an originally species rich extensively managed grassland (I) experiences intensification, then productivity increases, whereas richness decreases, resulting in a negative relationship (see side panel 1). Reducing land-use intensity from an intensively managed grassland (II), would decrease productivity, but not alter species richness (due to e.g. seed bank limitations), resulting in a less negative or neutral relationship (see side panel 2 and **a**). By actively reintroducing new species in a grassland with reduced land-use intensity (III), we would expect richness (as well as the gradient in species richness) to increase, concomitantly leading to increases in biomass production (due to e.g. species complementarity). This would result in a positive relationship between species richness and productivity (see side panel 3 and **b**). Note that the species richness gradient in panel III is longer than in panel I and II, because of the species pool enrichment through introduction of new species. *LUI* land-use intensity of grasslands. Blue two-sided arrows represent relationships between species richness and productivity. Green arrows indicate gradient in land-use (high: dark green vs low: light green). Red dashed line represents the hump-backed model (Grime et al. 1973).

As a result, this could lead to a negative relationship between species richness and productivity in managed grasslands, as indeed found in multiple studies (^16,24,25^, but see^26^). Given this, one would expect that if grassland land-use intensity is reduced, i) biomass production would decrease. At the same time, we would expect ii) species richness would remain low in the short term^12^, as seed bank limitations^27,28^, as well as dispersal limitations^29–31^, limit the recovery potential of species previously lost due to past intensive grassland land-use. As a result, with a reduction in grassland land-use intensity (e.g. reduced fertilization input, and reduced mowing/ grazing intensity), relationships between species richness and biomass production would be expected to shift from negative towards more neutral (Fig. 1, side panel 2 and a). Only after actively reintroducing plant species, positive relationships between species richness and biomass production could be expected due to increases in biomass production following species gains^12^ (Fig. 1, side panel 3 and b). While seed addition have been found to increase plant cover in some grasslands^32^, so far, previous studies have shown that sowing-induced increases in plant species richness into managed grasslands does not always lead to increases in biomass production (^33^, but see^34^). Furthermore, many of these studies were performed in unfertilized grasslands, and few tested the effects of seed addition on biomass production along a large gradient in grassland land-use^35^. Thus, studying the influence of sowing-induced increases in species richness on biomass production along a large gradient in grassland land-use intensity may advance our understanding about how species pool limitations affect biodiversity-productivity-relationships in managed grasslands. Ultimately, since high productivity in managed grasslands are an important part of climate mitigation strategies, illuminating these gaps is an important step in understanding how Biodiversity-ecosystem functioning (BEF hereafter) relationships scale.

Studies of biodiversity-productivity relationships in managed grasslands (e.g.^11,24^) so far only investigated one (e.g. reducing mowing frequency:^36^; sowing of new species:^11^) or two contexts (e.g. variation in grassland land-use intensity and seed addition:^37^) of the Schmid (2002) framework at the same time. However, we need a single study in which relationships between grassland species richness and biomass production are studied simultaneously in different contexts, such as proposed by Schmid (2002). Importantly, the shape of relationships between species richness and biomass production may also differ between seasons, because of seasonality in the availability of plant resources, such as soil moisture. Several studies suggest a higher efficiency of more diverse plant communities in partitioning resources, such as water^38,39^. Hence, one could expect stronger effects of species richness on biomass production in summer, when water is most limiting^40^.

In this study, we assess the effect of plant species richness on biomass production within grasslands managed at different intensities (by mowing, grazing and/or fertilization) in Germany and in both the spring as well as the summer season. Specifically, we aim to distinguish between relationships of species richness and biomass production (i) across grasslands varying in key components of grassland land-use intensity (i.e. fertilization, mowing and grazing) (Fig. 1, side panel (1), (ii) under ambient and reduced land-use intensity within grasslands (Fig. 1, side panel (2), and iii) in the latter settings, in which additionally, the variation in plant species pool size is experimentally enlarged through seed additions (Fig. 1, side panel (3). We performed our study in 150 managed grasslands across three regions in Germany, strongly varying in grassland land-use^41^. In 45 out of these 150 grasslands, land-use has been experimentally reduced, and new, native plant species that were previously locally absent (but present in the regional species pool), were sown. We hypothesize that (Fig. 1):

1. Due to underlying differences in grassland land-use intensity, there will be an overall negative correlation between species richness and biomass production in managed grasslands (Fig. 1, side panel 1).
2. When grassland land-use intensity is reduced, biomass production decreases, but species richness remains unaltered, more neutral relationship between species richness and biomass production (Fig. 1, side panel 2 and a).
3. Further, the addition of new seeds will increase species richness. This will result increased biomass production in sown compared to un-sown grasslands (Fig. 1, side panel b), causing an overall positive relationship between species richness and biomass production (Fig. 1, side panel 3).

## Results

### Effect of present land-use, reduced land-use, and seed addition on plant species richness and diversity

**Figure 2:**
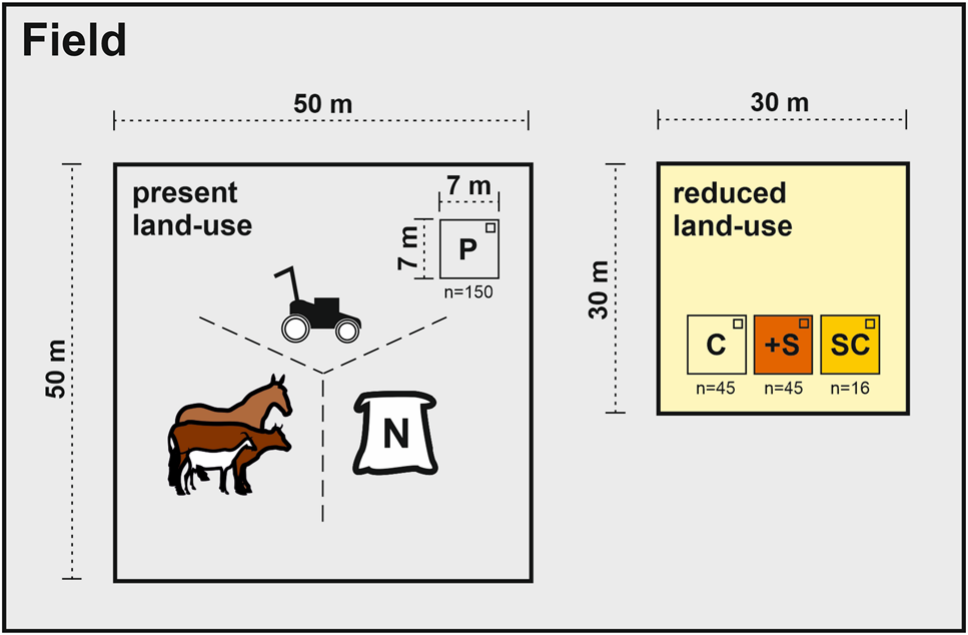
Overview showing the experimental design. In all three regions, 50 fields (150 in total) were selected, where present land-use varied in mowing frequency, grazing intensity and fertilizer input. Within those fields, a 50 × 50 m present land-use grassland site was established. Each present land-use grassland site contained a plot (7 × 7 m). All 150 present land-use plots (full set) represent the full gradient of land-use (high to low land-use intensity). In 15 fields (45 in total), out of the 150 fields, a 30 × 30 m reduced land-use grassland site was established in which land-use was reduced, meaning that these fields were only mown once a year, whereas grazing and fertilization were stopped completely. All background land-use plots which were located in the same field as an reduced land-use grassland site represent the subset of present land-use plots. All reduced land-use grassland site had two or three treatments (7 × 7 m treatment plots): a plot in which only land-use was reduced (C), a plot in which species richness was manipulated by scarifying the soil and seeding new species (+ S), and in some reduced land-use grassland site a third plot in which only soil scarifying but not seed addition took place (SC). In all plots a 1 × 1 m subplot was established, where we measured plant biomass, performed vegetations surveys and additional abiotic parameters (potential productivity). Thus, we collected data from 256 subplots in total across three regions in Germany. P: present land-use plot, C: reduced land-use—control, + S: reduced land-use + seed addition, SC: reduced land-use + soil scarifying.

**Figure 3:**
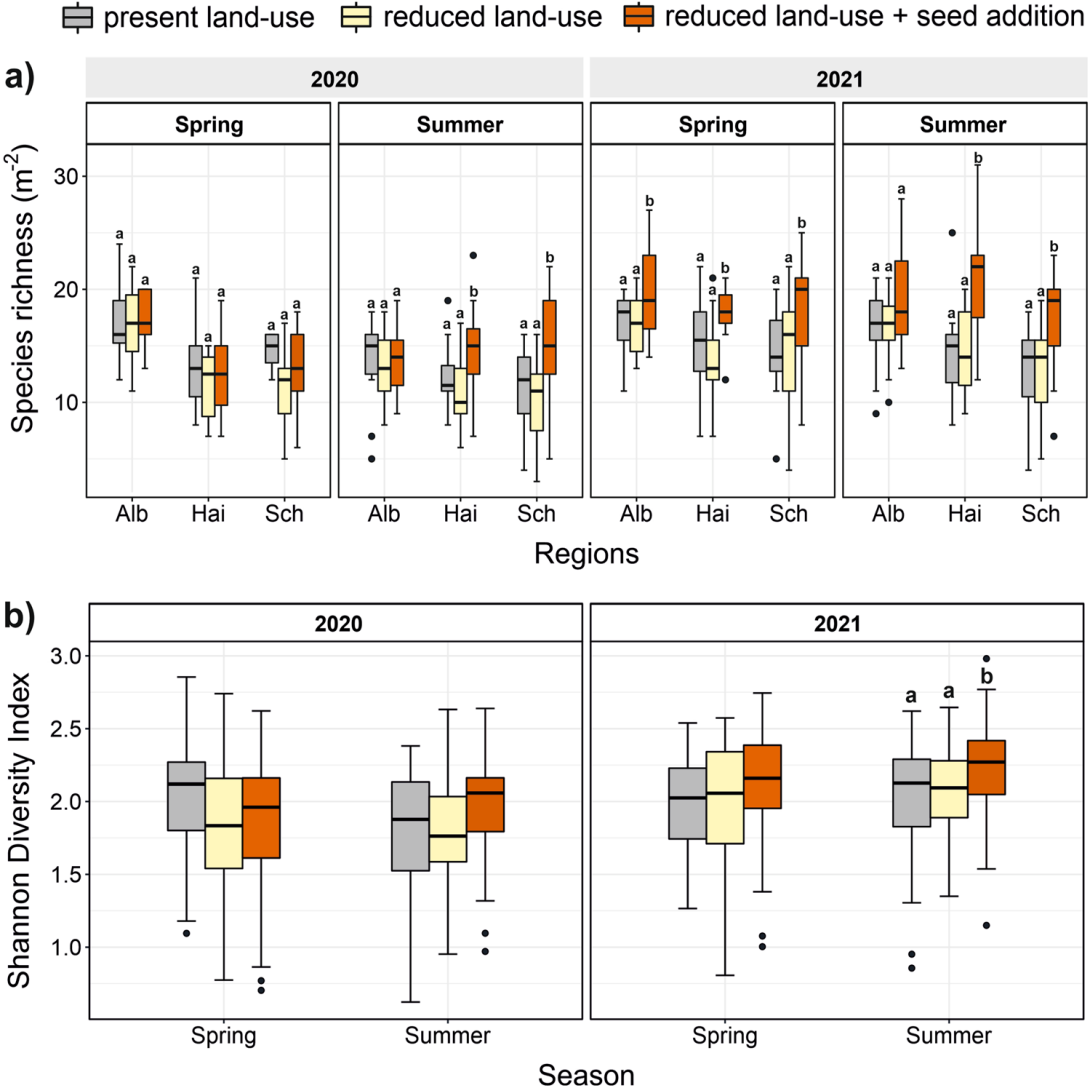
Response of (**a**) species richness (m^−2^) and (**b**) Shannon diversity to the present land-use subplots covering the reduced land-use intensity gradient (present land-use subplots located together in a field with a reduced land-use plot), reduced land-use and reduced land-use + seed addition, in three regions (Alb: Schwäbische Alb; Sch: Schorfheide-Chorin; Hai: Hainich-Dün). For Shannon diversity, no separation between regions, as treatment effects were consistent across regions. present land-use: grey; reduced land-use: yellow; reduced land-use + seed addition: red. Bars sharing a letter (**a**, **b**) do not differ significantly (*p* < 0.05, Suppl. Table S10).

Subplots with present land-use had similar levels of species richness and Shannon diversity to reduced land-use subplots (Fig. 3a,b, Suppl. Tables S1 and S7). Seed addition significantly increased species richness, from summer 2020 onwards in all regions, except Schwäbische Alb where only spring 2021 showed a significant effect (Suppl. Table S1). A strong increase in species richness as response to seed addition was observed in summer 2020 in the Schorfheide-Chorin (t_4,83.74_ = 3.20, *p* < 0.01) and in summer 2021 in the Hainich-Dün (t_4,85.38_ = 2.68, *p* < 0.01). After two years, species richness in reduced land-use with seed addition increased on average by five species in the Schorfheide-Chorin, six species in the Hainich-Dün, and two species in the Schwäbische Alb (in comparison to species richness in present land-use as well as reduced land-use subplots). Similarly, seed addition significantly increased Shannon diversity in all regions, although only in summer 2021 (t_2,87.29_ = 4.04, *p* < 0.01, Fig. 2b, Suppl. Table S7). This was also visible when also looking at the abundancies of sown new species in comparison to unsown species (Suppl. Table S21).

### Relationship between biomass produced and species richness in managed grasslands

Our most parsimonious models indicated that species richness was significantly negatively related to biomass produced, although in spring 2020 only in the Schorfheide-Chorin (Fig. 3, Suppl. Table S2). Strong significant negative relationships between species richness and biomass produced were observed in the Schorfheide-Chorin in spring 2020, and in spring 2021 across all regions (absolute value of the std. effect size below -0.3). Relationships were much weaker in the present land-use subplots with reduced land-use intensity gradient which were located in the same field as the reduced land-use subplots. Here we found weak relationships between species richness and biomass produced in both years, although in spring 2021 relationships were comparable to the present land-use subplots covering the full land-use intensity gradient (Fig. 3, Table 1). In spring 2020, the most parsimonious model also included a quadratic effect (richness effect: std effect size is 1.29; CI:  − 0.23, 2.81; F = 3.73; *p* = 0.07) in addition to a linear richness effect (std effect size is  − 1.09; CI:  − 2.62, 0.45; F = 3.01; *p* = 0.09), but since this was the only model in which a quadratic richness effect occurred, and since this effect was relatively weak and non-significant, in Table 1 we only put models without quadratic richness effects, for reasons of comparability.

**Table 1:** Relationships between species richness and biomass produced among different treatments and regions (Alb: Schwäbische Alb; Hai: Hainich-Dün; Sch: Schorfheide-Chorin). Effect sizes indicate the standardized effect of richness on biomass produced. Additionally shown are confidence intervals (CI), f-values (F) and *p*-values (P). Pres. LU: present land-use (full set: full land-use intensity gradient, subset: reduced land-use intensity gradient); Red. LU: reduced land-use, Red. LU + S: reduced land-use and seed addition; Δ Red. LU + S: based on Δ-richness and biomass produced values, i.e. richness/ biomass produced in Red. LU + S subplot minus richness/ biomass produced value in associated reduced land-use subplots. Effect sizes for which related CI’s are not crossing zero, and significant p-values are printed in bold. Although some of the most parsimonious models include a squared term of richness (always non-significant), we show only model without squared term to guarantee comparability between models (see Suppl. Fig. S2).

		Richness: standardized effect sizes				CI				DF	F	P
		*all*	*Alb*	*Hai*	*Sch*	*all*	*Alb*	*Hai*	*Sch*			
Pres. LU (full set)	*SP ‘20*	**–**	–0.12	–0.14	**–0.94**	–	[–0.46, 0.22]	[–0.83, 0.78]	[–1.54, –0.10]	1	4.17	**0.04**
	*SP ‘21*	**–0.40**	–	–	–	[–0.56, –0.23]	–	–	–	1	39.26	** < 0.01**
Pres. LU (subset)	*SP ’20*	0.20	–	–	–	[–0.07, 0.48]	–	–	–	1	2.77	0.11
	*SP ’21*	–0.34	–	–	–	[–0.70, 0.03]	–	–	–	1	3.61	**0.07**
Red. LU	*SP ‘20*	–0.22	–	–	–	[–0.63, 0.19]	–	–	–	1	0.71	0.41
	*SU ‘20*	**0.32**	–	–	–	[ 0.03, 0.62]	–	–	–	1	4.14	**0.05**
	*SP ’21*	0.00	–	–	–	[–0.40, 0.40]	–	–	–	1	0.00	0.99
	*SU ‘21*	0.25	–	–	–	[–0.10, 0.60]	–	–	–	1	2.15	0.15
Red. LU + S	*SP ‘20*	–	**–0.51**	**0.39**	0.34	–	[–0.88, –0.14]	[ 0.13, 1.67]	[–0.06, 1.75]	1	1.21	0.28
	*SU ‘20*	–	–0.01	0.36	0.34	–	[–0.38, 0.36]	[–0.40, 1.14]	[–0.41, 1.10]	1	2.40	0.13
	*SP ’21*	–	–0.14	–0.34	0.12	–	[–0.54, 0.26]	[–1.14, 0.74]	[–0.76, 1.28]	1	2.09	0.16
	*SU ‘21*	–	0.20	–0.21	0.02	–	[–0.57, 0.96]	[–2.08, 1.25]	[–1.52, 1.16]	1	0.32	0.57
Δ Red. LU + S	*SP ‘20*	0.07	–	–	–	[–0.26, 0.40]	–	–	–	1	0.20	0.66
	*SU ‘20*	0.17	–	–	–	[–0.16, 0.50]	–	–	–	1	1.12	0.30
	*SP ’21*	–0.24	–	–	–	[–0.55, 0.06]	–	–	–	1	2.59	0.12
	*SU ‘21*	0.16	–	–	–	[–0.16, 0.47]	–	–	–	1	1.02	0.32

### Relationship between biomass produced and species richness when reducing grassland land-use intensity and sowing new species

After reducing land-use intensity within grasslands, the strength and direction of the effect of species richness on biomass produced varied substantially between seasons, although effects were consistent across regions (Fig. 5a, Suppl. Table S3). Over all regions, strong (absolute value of the std. effect size exceeding 0.3) significant positive relationships between species richness and biomass produced were observed in summer 2020 after reducing grassland land-use intensity (Table 1).

When analysing both the reduced land-use subplots and the reduced land-use combined with seed addition subplots, our most parsimonious models again indicated that effects of species richness varied among seasons, but also among year and regions (interaction between species richness and region) (Fig. 5b, Suppl. Table S4).

Strong (absolute value of the std. effect size exceeding 0.3) significant positive effects of richness on biomass produced, when analysing the reduced land-use subplots and the reduced land-use with seed addition subplots, were found in the Hainich-Dün and in the Schorfheide-Chorin in spring 2020 and in summer 2020, while strong significant negative effects (absolute value of the std. effect size below  − 0.3) were only found in the Schwäbisch Alb in spring 2020 and in the Hainich-Dün in spring 2021 (Table 1). However, when analysing the relationships between the effective increase in species richness (delta between reduced land-use + reduced land-use with seed addition subplots) and the effective increase in biomass (delta between reduced land-use + reduced land-use with seed addition subplots), no significant relationships were found (Fig. 6, Suppl. Table S5).

In the sensitivity analysis, where we focused on those sites (Schwäbische Alb and Hainich-Dün) where (in addition to new seeds) unintentionally sown resident species were added, we found a strong (absolute value of the std. effect size below  − 0.3) significant negative relationship between species richness and biomass produced in the Schwäbisch Alb in spring 2020 (Suppl. Table S16). However, when only considering the Schorfheide-Chorin (only new seeds sown), we detected a marginally significant positive relationship between species richness and biomass produced (Suppl. Table S15). In the sensitivity analyses where we analysed the relationships between the effective increase in species richness and the effective increase in biomass, no relationship was found for neither, the combination of Schwäbische Alb and Hainich-Dün (seed mixture sown, Suppl. Table S18) nor the Schorfheide-Chorin (new seeds sown, Suppl. Table S17).

### Covariates altering the relationship between biomass produced and species richness

In all our models analysing the effect of species richness on biomass produced, we also corrected for potential covarying factors such as soil moisture, potential productivity (proxy for soil fertility) and sampling date (as Julian date). We found that potential productivity was an important covariate affecting biomass produced, although we observed both negative and positive correlations. Similar, sampling date was an important covariate positively affecting biomass produced (Suppl. Tables S2, S3 and S4). In contrast, despite being included in our most parsimonious models, soil moisture did not show any strong associations with biomass produced (Suppl. Tables S2, S3 and S4). Furthermore, when analysing the effects of species richness on biomass produced in reduced land-use subplots and reduced land-use + seed addition subplots, we included grassland land-use intensity (LUI) as additional covariate. However, although LUI was included in most of our most parsimonious models, we did not observe any strong associations with biomass produced (Suppl. Tables S3 and S4).

## Discussion

We investigated relationships between plant species richness and biomass produced across different contexts in for central Europe characteristically managed grasslands (fertilized, mown and/or grazed, see^14^), based on the theoretical framework of^12^ (2002). Our results showed that in line with expectations, relationships between species richness and biomass produced were predominantly negative in managed (by fertilization, mowing and/or grazing) grasslands. In contrast, when reducing land-use intensity, relationships between species richness and biomass produced shifted from negative towards neutral. However, contrary to our hypothesis, seed addition in grasslands with reduced land-use intensity did not cause positive relationships between plant species richness and biomass produced.

**Figure 4:**
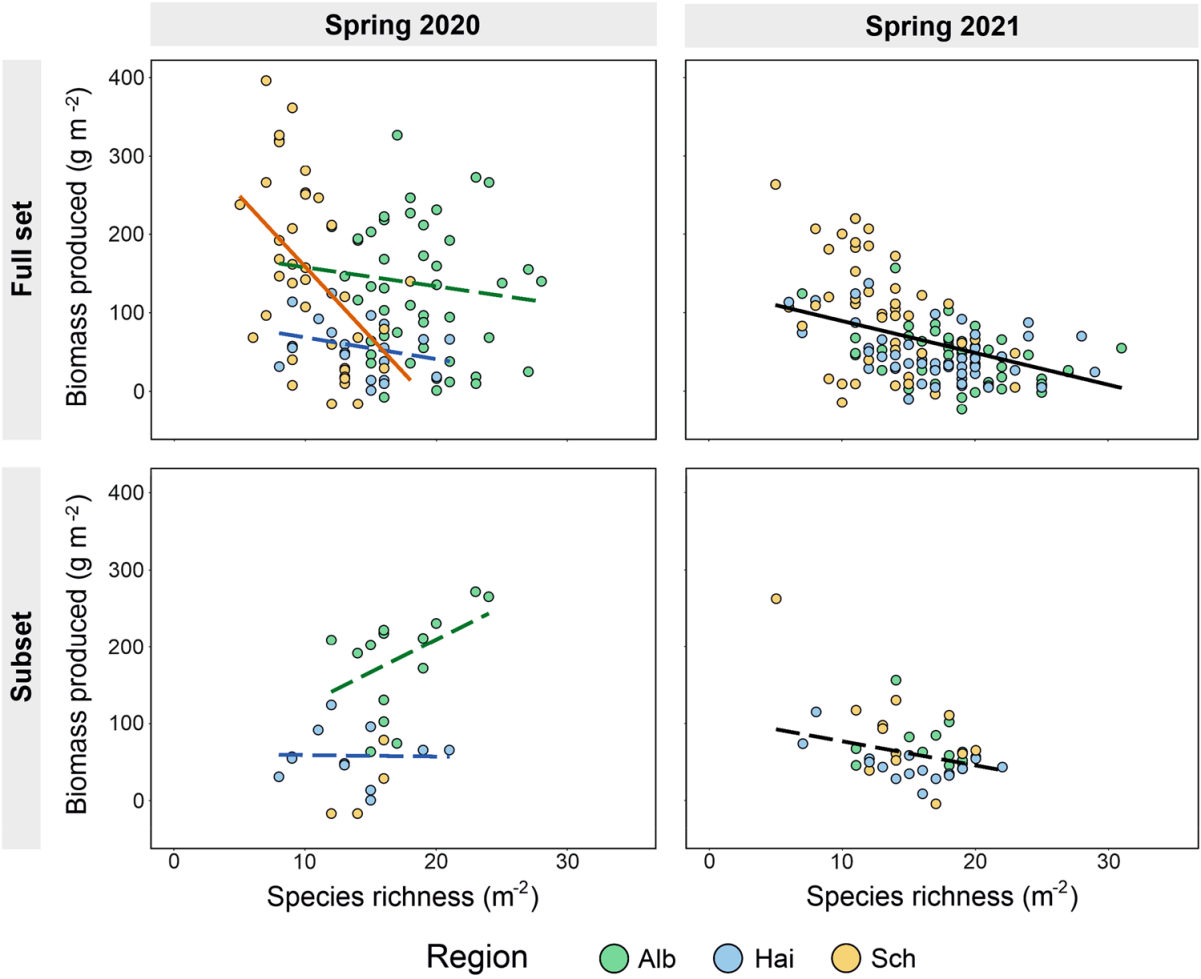
Biomass produced (g m^−2^) plotted against species richness for the present land-use subplots covering the full land-use intensity gradient (upper panels) as well as for those present land-use subplots covering the reduced gradient in land-use intensity located together in a field with a reduced land-use treatment (lower panels) for spring 2020 and 2021, colour coded for all three regions (Alb: Schwäbische Alb; Sch: Schorfheide-Chorin; Hai: Hainich-Dün). Regression lines are shown for each region, and are dashed when not significant (see Suppl. Table S2). If effects of species richness on biomass produced did not differ between regions, the overall regression is shown in black, and dashed when not significant. Due to low data coverage, no regression is shown for the present land-use subplots covering the reduced gradient in land-use intensity in the Schorfheide-Chorin in spring 2020. Although some of the most parsimonious models include a squared term of species richness (always non-significant), we show only modelled relationships of models without squared term to guarantee comparability between models.

Our first key result was that species richness and biomass produced were negatively related in managed grasslands when correcting for covarying environmental factors, although in the first year only in the Schorfheide-Chorin. This result is in line with our hypothesis (Fig. 1), as well as with findings from several other studies (^16,24,25,42^, but see^26^). It is likely that this negative relationship is mainly driven by grassland land-use, in particular by elevated nutrient inputs due to fertilization, that has been shown to negatively affect plant diversity, while promoting biomass production^21,43^. In addition, mowing frequency as well as grazing intensity have also been found to promote biomass production in some cases, while decreasing species richness^15^. Of all regions, the Schorfheide-Chorin showed the strongest negative relationship. The Schorfheide-Chorin is characterized by drained peat soils, especially rich in organic matter^41^. Furthermore, the former drainage of peat soils has been shown to be associated with increased nutrient availability driven by enhanced mineralization^44^. Hence, increased nutrient availability may have positively affected biomass production in the Schorfheide-Chorin, while simultaneously decreasing plant diversity^21,43^, explaining the strong negative relationship. In contrast, the overall negative relationship between species richness and biomass produced was much weaker in the grasslands covering the reduced gradient in land-use, which were in the same field as plots where land-use was experimentally reduced. Especially in spring 2020, the relatively weak relationships between biodiversity and productivity were potentially attributed to missing data points, in particular from typically high productivity but species poor plots in the Schorfheide-Chorin, potentially not allowing to detect stronger negative relationships. However, in spring 2021 relationships were comparable to the present land-use subplots covering the full land-use intensity gradient (Fig. 4). One possible explanation might be that, in the present land-use plots covering the reduced land-use intensity gradient we were missing plots from both ends of the land-use intensity gradient (low and high), which limited the strength of opposing effects of grassland land-use intensity on species richness versus biomass production. Thus, these mostly intermediate intensively used plots potentially did not allow to detect strong patterns. Another explanation for the relatively weak negative relationship between species richness and biomass production even within the present land-use subplots covering the full land-use intensity gradient is that we measured biomass produced only in spring (in may), and not at peak biomass (in July/August). Thus, relationships might be expected to be more negative in summer, when effects of land-use intensity are expected to be strongest.

**Figure 5:**
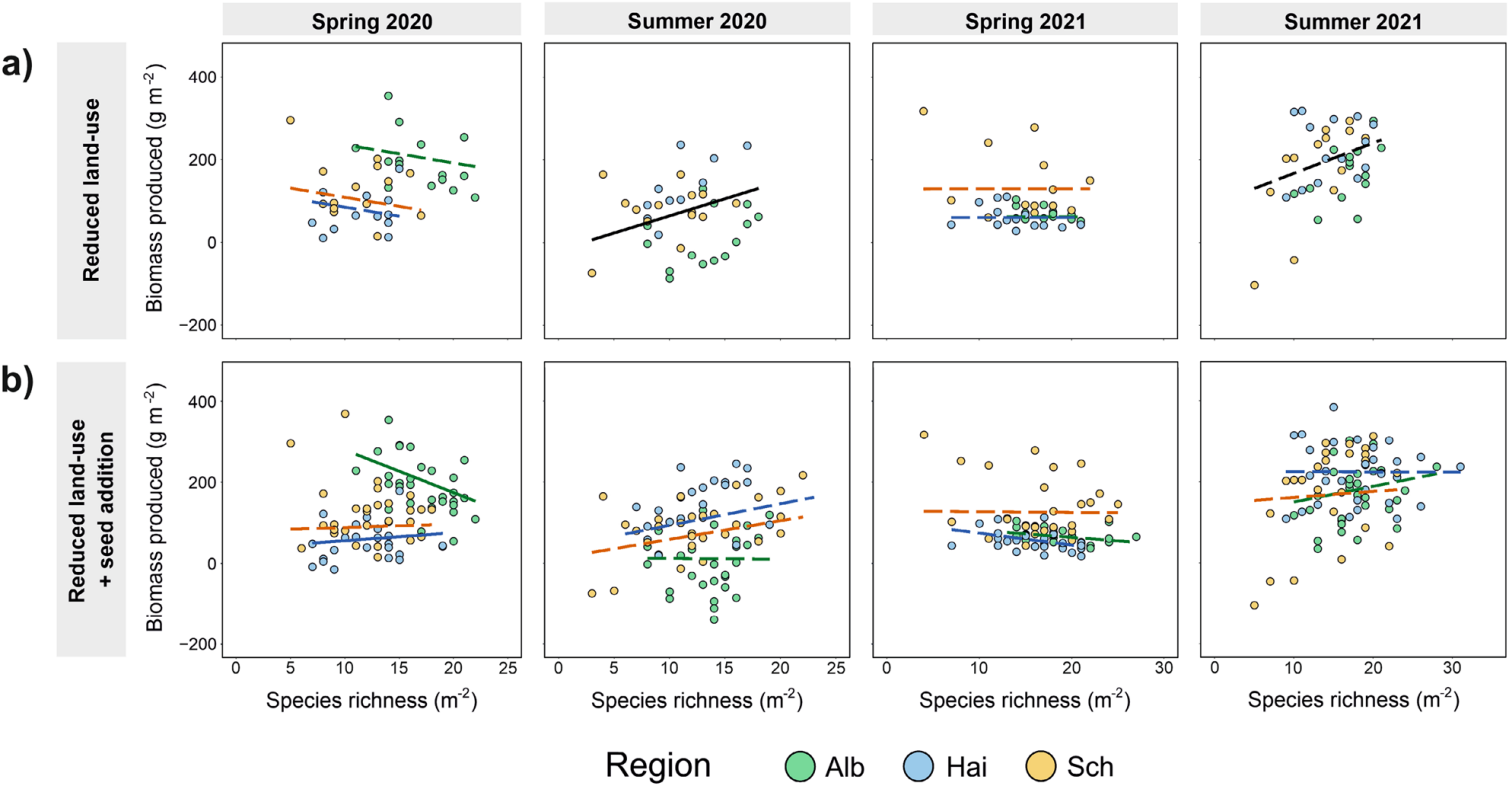
Biomass produced (g m^−2^) plotted against species richness for (**a**) the reduced land-use treatment and (**b**) for the reduced land-use treatment as well as the plots reduced land-use with seed addition, for all three regions (Alb: Schwäbische Alb; Hai: Hainich-Dün; Sch: Schorfheide-Chorin) and for spring and summer 2020 and 2021. Regression lines are shown for each region separately (when interaction between species richness and region was found), and are dashed when not significant (see Suppl. Tables S3 and S4). If effects of species richness on biomass produced did not differ between regions, the overall regression is shown in black, and dashed when not significant. For further information on effect sizes of species richness on biomass produced for the different treatments see Suppl. Fig. S4. Although some of the most parsimonious models include a squared term of species richness (always non-significant), we show only modelled relationships of models without squared term to guarantee comparability between models.

In line with our hypothesis two, we found that the negative trend in the relationship between plant species richness and biomass produced observed in spring 2021 disappears (and became neutral), when grassland land-use intensity was reduced and confounding environmental factors (such as present land-use, soil moisture, sampling date and potential productivity) were accounted for (Fig. 5a). As expected, land-use reduction did not alter species richness, however, contrary to our expectations, neither did land-use reduction lead to strong decreases in biomass produced. Although the average biomass produced did not change when reducing grassland land-use intensity, levels of biomass produced did change in individual plots, resulting in a change of the slope for the overall relationship. This might be due to the stop of fertilization, leading to a reduction in productivity specifically in less diverse communities^45^. One possible explanation for the lack of significant response of biomass produced to reducing land-use might be, that effects of former land-use, such as residual high nutrient loads, take more years to fully disappear after cessation of fertilization^46^. The lack of a recovery of species richness after land-use reduction is potentially due to soil seed bank depletion seen in many grasslands^27,28^, as well as low colonization probability from surrounding areas^29–31^, and negative impacts of former land-use^31,47^. When comparing the relationships between species richness and biomass produced in spring and summer after a reduction in grassland land-use intensity, relationships differed substantially between seasons. In the summer of the first and the second year after land-use reduction, we found positive relationships across all three regions (Fig. 5a). One possible explanation for this result might be the varying importance of environmental factors, such as soil moisture, across seasons. Diversity is suggested to increase partitioning of nutrients, light, and water among species^38,39^. Hence, plant diversity might be most important for biomass production in the summer season when water is most limited. In this season, species differences in space use (e.g. due to allocation of roots to different depths, see^40^), can result in an increased total water uptake. However, since we lack data on biomass produced in present land-use plots measured in summer of either 2020 or 2021, we do not have a fair comparison to the reduced land-use treatments. Thus, we cannot rule out that the observed seasonal variability in the reduced land-use treatments may also be present in the present land-use plots. Furthermore, to fully understand the seasonal variation in the relationships between species richness and biomass produced, further investigation with longer term observation are needed.

**Figure 6:**
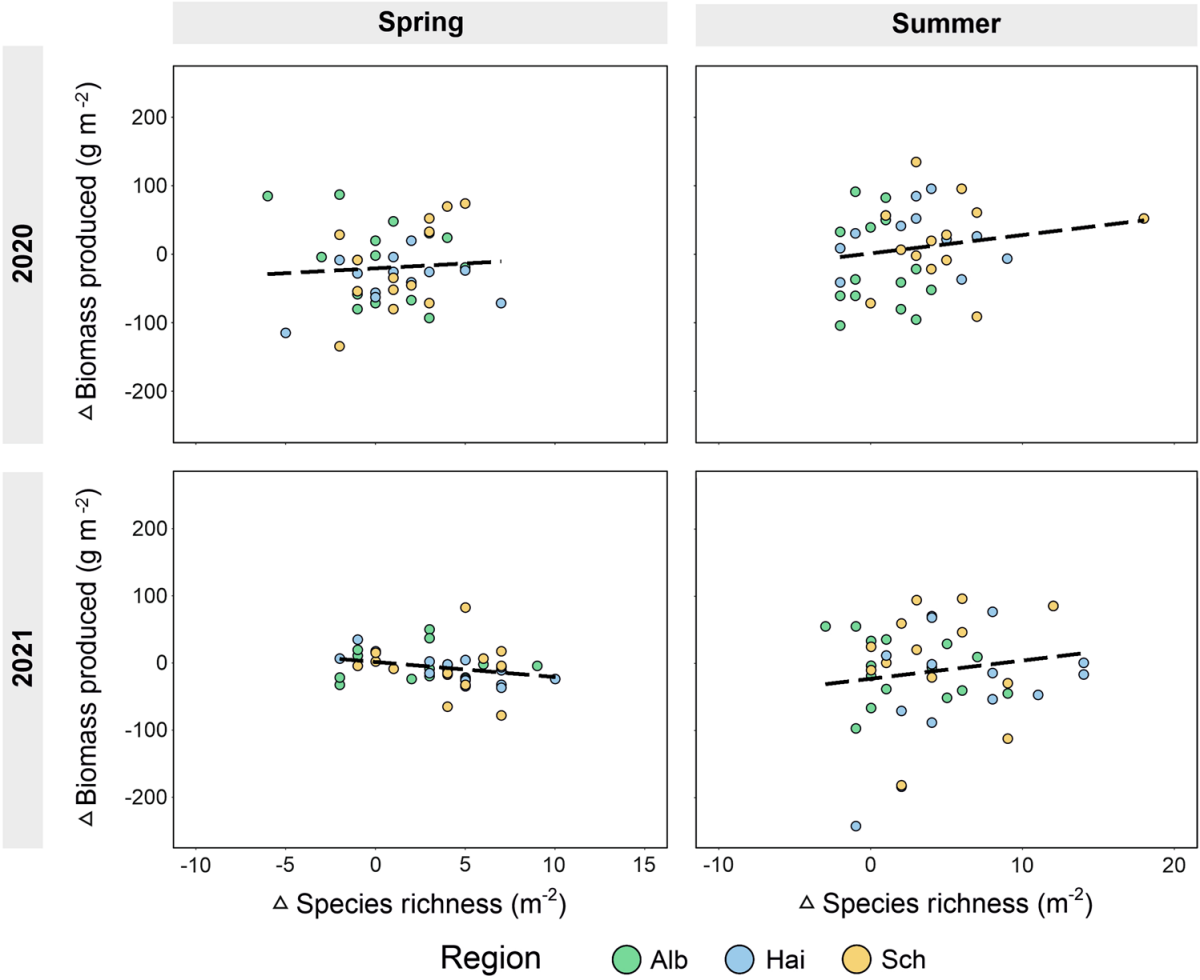
Delta values (between reduced land-use + seed addition and reduced land-use subplots) for Biomass produced (g m^−2^) plotted against species richness colour coded by regions (Alb: Schwäbische Alb; Hai: Hainich-Dün; Sch: Schorfheide-Chorin). Regression lines represent significant correlations (black line). The overall regression (if no regional differences were observed) between biomass produced and species richness is shown in black, and dashed when not significant (see Suppl. Table S5). Although some of the most parsimonious models include a squared term of species richness (always non-significant), we show only modelled relationships of models without squared term to guarantee comparability between models.

Similar to previous studies^11,35,37^, and as hypothesized, we found that seed addition of new species significantly increased species richness and Shannon diversity in all three regions (Fig. 3a, b). Prior to seed addition, we scarified the upper soil layer to enhance seedling establishment^35,37,48,49^. However, soil scarifying itself did not have any effects on species richness (Suppl. Table S8), so that we are confident that observed species gains were mainly driven by the seed addition treatment. Contrary to our hypotheses (Fig. 1), the seed addition treatment did not lead to a clear increase in biomass produced, and gains in species richness were not associated with increases in biomass produced (Fig. 6). Thus, we only observed positive relationships between species richness and biomass produced when reducing land-use and adding new seeds in spring 2020 for the Hainich-Dün, and marginally significant in the Schorfheide-Chorin, while no clear relationship was found in either summer 2020, or in 2021 (Fig. 5b). This is contrary to our hypothesis, in which we expected that a widening of the species richness gradient would also lead to gains in biomass produced, and begs the question why biomass produced did not respond in a similar way as species richness to seed addition. We identify four possible explanations. First, BEF-relationships are often asymptotic in grassland experiments^50^, with biomass gains levelling off when richness levels exceed approximately 10 species, possibly due to a saturation in resource use^8,51^. Only 8.7% of our managed grassland plots had less than 10 species, making them more diverse than most grassland communities in biodiversity experiments^52^. This might explain why there was little scope for strong relationships between species richness and biomass produced. However, we did additionally test for non-linearity between species richness and biomass produced (by including the squared term of species richness in our models), but we did not observe any significant quadratic richness effects. Nevertheless, the limited gradient in species richness across our studied grasslands might have precluded the detection of strong non-linear relationships. A second possible reason for a lack of response in biomass produced after seed addition, is that increases in species richness might have been too low to induce responses in biomass produced. In our study, average richness gains varied from 2.3 to 6.5 species across regions, which is comparable to other studies (Ladouceur et al. 2020; Freitag et al. 2021). While strongly significant, it might be that these increases in species richness were still too moderate to lead to increases in biomass produced. A third possible explanation for a lack of stronger, positive BEF-relationships after seed addition is that species which successfully established after seed addition were still relatively low in abundance. However, we found that seed addition not only increased species richness but also Shannon diversity (in summer 2021) (Fig. 3a,b, Suppl. Table S7), as newly established species also had relatively high abundancies. Finally, it is possible that positive relationships between biomass produced and species richness will emerge in the longer term, which has also been shown in a study by Bullock et al.^11^ and Pichon et al.^34^, where positive effects of seed addition on biomass production were increasing over time following seed addition. However, it might also be that differences in seed addition treatments (seed mixture of both resident and previously absent species in Hainich-Dün and Schwäbische Alb; only seeds of previously absent species the Schorfheide-Chorin) could explain some of the differences in relationships between biomass produced and species richness when reducing land-use and adding new seeds. Although a sensitivity analysis revealed overall no strong differences between relationships when accounting for applied seed mixtures (Suppl. Tables S15 and S16), we cannot fully rule out that regional conditions in how treatments were applied mediated the effect of seed addition on biomass produced, and caused that the relationship was somewhat more positive (marginally significant in spring 2020) in the Schorfheide-Chorin where only new seeds were added.

The seemingly contrasting relationships between species richness and biomass production observed in managed and experimental grasslands led to an ongoing debate about the strength and relevance of BEF-relationships in managed systems^7–9^. Theoretical frameworks, such as the framework introduced by^12^ (2002), offer a possible explanation for the high variability of relationships observed in those managed systems. To our knowledge, our study is the first to thoroughly test the predictions of this framework in one single study, comparing the relationships between species richness and biomass production in different contexts in managed grasslands. In particular, although our study was not minimizing variation in abiotic factors or management compared to conventional grassland experiments, our results advance our knowledge about the relationships between species richness and biomass produced in managed grasslands. We demonstrated that the overall negative relationship between species richness and biomass production in managed grasslands can be neutralized, and even shifted towards positive relationships in specific cases, when reducing grassland land-use intensity. Although seed addition of new species did not lead to consistently positive relationships between species richness and biomass production in the short term, it is possible that in the long-term such relationships could emerge, as many studies reported increasing effects of biodiversity over time (^53^,^50^). Moreover, the effects of former land-use may take longer to disappear^46^ which might strengthen positive relationships between plant diversity and biomass production in the long run. Finally, we observed strong positive relationships between species richness and biomass production specifically in summer, suggesting a varying importance of species richness on biomass production depending on the relative importance of environmental factors (such as soil moisture).

In summary, our findings suggest that under certain conditions, positive relationships between species richness and biomass production are possible in managed grasslands. However, when grasslands vary in land-use intensity, such relationships are absent or even negative. Although we did not find clear evidence that increases in the species pool altered relationships between species richness and biomass production, further research, is needed to fully parse the interactions between dispersal limitation and BEF-relationships in managed grasslands. Furthermore, due to some missing data particularly in the first year of the study (due to corona related restrictions) the results of this study should be considered early-stage, and hence, future studies with longer-term investigation are needed. Investigating the relationships between species richness and biomass production in different contexts and by combining observational and experimental approaches will advance our understanding of seemingly contrasting BEF-relationships in managed grasslands, which ultimately help us to inform management strategies for biodiversity conservation.

## Material and methods

### Study area

The study was conducted in 150 managed (fertilized, mown and/or grazed), largely unsown (142 unsown vs 8 sown for previous land improvement) fields located in the three German regions (50 in each region) of the Biodiversity Exploratories project (www.biodiversity-exploratories.de; Fischer et al. 2010). These three regions are the UNESCO Biosphere Reserve Schwäbische Alb (Swabian Jura) (48° 43′ N, 9° 37′ E) in the south-west, the National Park Hainich-Dün (51° 20′ N, 10° 41′ E) in the centre and the UNESCO Biosphere Reserve Schorfheide-Chorin (53° 02′ N, 13° 83′ E) in the north-east of Germany, differing in climate and soil characteristics. Thus, the Schorfheide-Chorin is characterised by high temperatures and deep fertile soils, while the Schwäbische Alb by high precipitation and shallower phosphorus poor soils (see^41^ for more details on regional differences). All fields were agriculturally managed as meadows, pastures, or mown pastures (mown once per year either before or after they were grazed), spanning a gradient of grassland land-use intensity (present land-use hereafter), productivity and plant diversity^14,41^ (for further information on regional, seasonal and yearly means see Suppl. Table S9). Land-use intensity in these selected grasslands is a composite measure of mowing frequency (cuts year^−1^, mean: 0.99, max: 4.09), grazing intensity (Livestock units * d ha^−1^ year^−1^, mean: 1, max: 10.96) and amount of fertilization (kg N m^−3^ year^−1^, mean: 0.99, max: 8.38) (for regional means see Suppl. Table SS14). We deliberately studied grassland diversity and biomass production in managed grasslands along a gradient in land-use intensity, as we aimed to investigate how land-use reductions in combination with increases in species richness via seed addition may influence BEF-relationships in managed grasslands (in contrast to conventional experiments which minimize variation in grassland management). Although temporal variation in land-use is also an important driver of species richness in managed grasslands^54^, we specifically focus on spatial variation as temporal variation of land-use in our studied period was relatively low (Pearson correlation between yearly LUIs on average > 0.8). All studied grasslands belong to mesic Arrhenatherion elatioris W. Koch 1926 and Cynosurion cristati Tx. 1947 communities, depending on soil moisture as well as altitude^43^. The 10 most abundant plant species occurring in all regions were: *Poa pratensis* L., *Lolium perenne* L., *Dactylis glomerata* L., *Alopecurus pratensis* L., *Festuca rubra* L., *Taraxacum sp.* F.H.Wigg., *Arrhenatherum elatius* L., *Festuca pratensis* Huds., *Phleum pratense* L., *Elymus repens* L.

### Experimental design

In each region, we studied the effect of plant species richness on biomass production in an observational setting, by reducing land-use intensity, as well as by manipulating species richness via seed addition. In 150 managed grasslands across the regions we established a permanent present land-use plot of 7 × 7 m in autumn 2019 (Fig. 2). As these 150 present land-use plots are spanning a large gradient in land-use (low to high land-use), we hereafter refer to these plots as: “full land-use intensity gradient”. Additionally, in 45 out of the 150 fields (15 per region) we established another permanent grassland site in autumn 2019 in which land-use was reduced (Fig. 2) to mowing only once a year in August or September (i.e. usually less than in adjacent present land-use plots) and no grazing or fertilization. Specifically, we applied a reduced mowing regime (more homogeneous land-use in comparison to grazing, which is more patchy), to maintain the grassland system (e.g. preventing shrub encroachment)^55^, as well as to imitate management typical for extensively managed grasslands in Europe. As these reduced land-use grassland sites were adjacent to a subset of present land-use plots, representing only a part of the full land-use intensity gradient of present land-use, we hereafter refer to those present land-use plots as: “Reduced land-use intensity gradient”. Specifically, we deliberately selected grasslands originally managed at moderate to high intensities to represent the Reduced land-use intensity gradient, as only a reduction in land-use within previously more intensively used grasslands enabled us to test the hypothesis proposed by Schmid^12^, and helped to avoid incorrect conclusions due to correlations between land-use intensity and covarying, environmental factors (e.g. soil texture or elevation) that exist in non-experimental settings.

Within each reduced land-use grassland site there was a plot of 7 × 7 m (reduced land-use plot hereafter) and in addition, we established another plot, in which species richness was experimentally manipulated (reduced land-use + seed addition plot hereafter) (Fig. 2). This was done by scarifying the reduced land-use + seed addition plot, whereby scarifying is a minor manual mechanical disturbance of the top soil surface down to 5 cm using lawn scarifier to increase seedling establishment by promoting seed-soil contact as well as by reducing light competition^37,48,49,51^, and then sowing new species (twice: in autumn 2019 and spring 2020, ranging from 40 to 74 species depending on deviance of the species pool at the site to the regional species pool). In the Schorfheide-Chorin region, only species originally absent in the plot (but present in the region) were sown, while in Hainich-Dün and the Schwäbische Alb, in addition to new species, unintentionally also species already present at the site were sown. In 16 out of the 45 reduced land-use plots, we also added a third plot, which was scarified once (just like in the reduced land-use + seed addition plots), but without sowing any new species (reduced land-use + soil scarifying plot hereafter). As soil scarification had no significant effect on plant species richness in the spring 2020 (Suppl. Table S8), and, as longer-term scarification effects are unlikely and typically decrease over time^35^, we did not collect data from reduced land-use + soil scarifying plots after spring 2020. In each plot, we established a 1 × 1 m subplot, where we monitored plant biomass and species composition. In total, we studied 150 present land-use subplots, as well as 45 reduced land-use subplots, 45 reduced land-use + seed addition subplots and 16 reduced land-use + soil scarifying subplots (256 subplots in total). However, in spring 2020 we were only able to sample 30 out of the 45 present land-use plots, due to management restrictions that prohibited plot access and logistic constraints due to the Covid-19 pandemic (for number of plots per treatment see Suppl. Table S9). All treatments within the same field were randomized, to reduce block effects.

### Data collection

All data was collected in the spring and summer (at peak biomass) season (April to May and July to August respectively) in 2020 and 2021. At each subplot, we estimated total vegetation cover (in percent) as well as the cover of each vascular plant species present. Furthermore, in each subplot we estimated standing biomass as the mean of four measures per subplot using a rising plate meter (Jenquip Manual Plate Meter). Biomass estimations were calibrated based on data from two additional 1 × 1 m subplots (100 calibration subplots per region, 300 in total) in each present land-use plot (Suppl. Table S15). In these, in spring 2021 both biomass estimations using a rising-plate meter (following the procedure described above) and standard biomass measurements (cutting and drying aboveground vegetation) were performed. Aboveground vegetation was cut to 4–5 cm height, dried at 80°C for 48 h and weighed (for further information see^56^). We also quantified present land-use intensity (LUI hereafter) for each of the years 2017–2019 (before our experiments were set up), as a composite measure of three land-use components: mowing frequency (number of cuts year^-1^), grazing intensity (density of livestock units per days of grazing ha year^−1^) and amount of fertilizer (kg N ha^-1^ year^-1^), based on^14^, using the LUI calculation tool^57^ implemented in BExIS (http://doi.org/10.17616/R32P9Q). Data on different land-use components were derived from yearly surveys of farmers and land owners (for more information see^58^). For more details on the quantification of LUI, we refer to^14^. Data on soil moisture at 10 cm depth was obtained from the Biodiversity Exploratories internal climate database, derived from permanent weather stations installed at each field (see Biodiversity Exploratories Instrumentation Project—BExIS dataset ID 24,766, Suppl. Table S15). As an additional covariate we further quantified the potential productivity of each plot as a proxy for soil fertility by performing a phytometric assay^59,60^. To do so, we collected two soil samples from each plot and cultivated the phytometer species *Taraxacum officinale* Kirschner H. Øllg. & Štěpánek on each soil sample in a greenhouse experiment. After four weeks of cultivation, we harvested all aboveground biomass by clipping it to ground level. Harvested biomass was then dried at 80°C for 48 h and weighed. Finally, the measured aboveground plant biomass representing the potential productivity at each plot was taken as a proxy for soil fertility (for further Information on the method see Appendix, “Supplementary methods”).

### Data processing and calculations

All taxonomic names were standardized using the package “Taxonstand” (^61^, version 2.2), after which we quantified species richness and the Shannon Diversity Index^62^ in each subplot. To calculate standing biomass (g m^-2^), we first did a calibration using data of rising-plate meter (averaged per subplot) and standard standing biomass measurements from the same subplot (2 × 150 calibration subplots in all present land-use plots, 1 × 1 m each). We did so by first performing a linear model with standard standing biomass (g m^-2^) as response variable and rising-plate meter measurements (0.5 cm increments) as predictor variable. We also performed an alternative model testing for non-linear relationships using squared plate meter measurements, however, based on the AIC^63^, the linear model was selected as the most parsimonious model (AIC for model with only linear term: 2942.98, model with squared term: 2959.72). We excluded data from five calibration subplots, due to measurement errors. The calibration model explained 83.22% of the variance in standard standing biomass (t = 37.94, *p* > 0.01, RSD = 38.85 g m^−2^, Suppl. Fig. S3, Suppl. Table S13). Based on this calibration model, we converted plate meter measurements in standing biomass (for further information see^56^). A possible limitation is that the data underlying this calibration was only collected in spring 2021. While this may have affected the biomass estimates in other seasons, it is not likely that it has led to systematic biases in biomass estimated that correlate with species richness. Biomass produced (here used as measure for net primary productivity) in spring was considered equal to standing biomass in spring. Offtake due to grazing animals or mowing was not included in the calculation, as most fields were mown/grazed at the end of the previous growing season, and not mown and hardly grazed before our biomass measurements took place (except in 4.7% of our studied plots, which were grazed before biomass measurements). Biomass produced in summer was quantified as the standing biomass in summer minus the standing biomass in spring. As we only studied fenced plots in the summer season, these were not influenced by grazer activities. Additionally, to obtain a mean LUI value over the time before our experiment was set up (2017–2019), LUI was averaged across time (see Suppl. Table S14). Furthermore, as potential covarying environmental factor we calculated the average soil moisture in spring (average across March, April and May) and summer (average across June, July and August) before vegetation surveys took place, for both 2020 and 2021.

### Data analysis

All data analyses were performed using R v. 4.1.1^64^. Statistical analyses were performed using *lme4*^65^, *lmerTest*^66^, *stats4*^64^, *multcomp*^67^, *car*^68^ and *MuMIn*^69^ packages. To study the effects of present land-use (present land-use plots—full land-use intensity gradient /reduced land-use intensity gradient) as well as different treatments (reduced land-use, reduced land-use + seed addition) on species richness and biomass produced (Hypotheses 2–3, Fig. 1a,b), we performed linear regressions (type III) including treatment and region as fixed effects, and for testing hypothesis 3, linear mixed effect models including treatment and region as fixed but also field as a random effect (to correct for field effects). We tested hypothesis 3 by including data from both reduced land-use and reduced land-use + seed addition plots, to increase the gradient in observed species richness enabling us to detect stronger relationships between species richness and biomass produced. We deliberately performed all above described models separately for each season (spring and summer) and year, as slightly different measures for productivity were used in spring (where productivity is equal to standing biomass) and summer (where productivity is calculated as biomass increments between spring and summer, by accounting for biomass removed by mowing or grazing), making models based on combined spring and summer measurements inappropriate. Furthermore, to test Schmid’s (2002) framework we deliberately sampled a large pool of grasslands managed at different land-use intensities to test whether relationships between biodiversity and biomass produced are more negative when a large gradients in land-use intensity exists. To test for non-linear relationships between species richness and biomass produced, we additionally included a squared term of species richness in all models (see^70^). Additionally, to statistically correct for the confounding effects of covarying factors we included present LUI (mean of 2017–2019), soil moisture, potential productivity (as proxy for soil fertility) and date of sampling campaign (as Julian date) as additionally covariates. Hence, the full model included species richness, region and additionally covariates as fixed effects, while in models testing hypothesis 3, also field as random effect was included. Since the inclusion of multiple covariates in models can cause multicollinearity, we omitted some variables based on variance inflation factor (see the next paragraph for more details). Pairwise comparisons between treatments were tested by performing a Tukey test using the function ‘emmeans’ from the package ‘emmeans’^71^. Since prior to the seed addition, the top soil layer was scarified (minor disturbance), we also tested whether soil scarifying itself had an effect on species richness, by performing a linear mixed effect model with data from spring 2020 only (as only in that season data from reduced land-use/ soil scarifying plots were collected), with treatment and region as fixed effect, and field as a random effect (to correct for field effects).

We studied the effects of species richness on biomass produced for different contexts: within present land-use plots (full land-use intensity gradient/reduced land-use intensity gradient) (Fig. 1, Hypothesis 1), within reduced land-use plots (Fig. 1, Hypothesis 2), as well as among both the reduced land-use and reduced land-use + seed addition plots, where the gradient in species richness was maximized (Fig. 1, Hypothesis 3), and for both spring and summer season. When statistically comparing the effects of present land-use with reduced land-use plots, we always used the present land-use with reduced land-use intensity gradient to guarantee fair comparison among treatments. Furthermore, we also assessed how the change of species richness due to seed addition was related to changes in biomass produced (Fig. 1, Hypothesis 3). To do so, we calculated delta species richness and biomass produced values, by subtracting values of species richness and biomass produced measured in reduced land-use plots from the species richness and biomass produced values from corresponding (i.e. from the same field) seed addition plots. We then performed a linear regression including delta species richness, a squared term of delta species richness (to test for non-linear relationships, see^70^) and region as the only predictor variables of delta biomass.

For all models we performed a forward model selection procedure based on either a linear regression or a linear mixed effect model. However, stepwise selection was only applied on covariates (i.e. soil moisture, potential productivity, sampling date, present LUI) potentially effecting biomass produced. In particular, while being potential important predictors of biomass produced, we deliberately applied an exploratory analysis (i.e. stepwise selection) on the covariates, as we did not hypothesise a clear direction of the relationships between biomass produced and the respective covariates. Importantly, even when not being part of the most parsimonious model, we always included species richness, as this factor was, unlike e.g. covariates related to abiotic conditions, central to the hypotheses of our paper. Specifically, we did a stepwise forward selection adding predictors that (i) did not increase the AIC and (ii) did not lead to exceeding a variance inflation factor (VIF) of 3 (using the ‘vif’ function from package ‘car’, vif factor according to^72^), allowing us to select the most parsimonious model^63^ while avoiding multicollinearity between predictors. If species richness and region were both part of the most parsimonious model, we additionally tested (based on AIC) for the need to include an interaction between both factors. In contrast, quadratic richness effects were only included if reducing the AIC in our final model. We did not test for interactions between quadratic species richness effects and region, as we did not expect any such effects when formulating our hypotheses. When analysing the effects of species richness on biomass produced in the present land-use plots (full land-use intensity gradient /reduced land-use intensity gradient), we only analysed data from spring 2020 and 2021, as biomass produced was challenging to quantify for the summer season in present land-use plots due to mowing and/or grazing activities that took place in most of the plots after the spring field season.

Due to unintentional differences in seed addition treatments among regions (in the Schorfheide-Chorin: only new species sown vs in the Schwäbische Alb and Hainich-Dün: mixture of new species and resident species sown), we additionally conducted a sensitivity analysis reproducing all analyses testing the relationships between species richness and biomass produced when new seeds were sown (reduced land-use + seed addition plots), separately for only (1) the Schorfheide-Chorin (only new species) and (2) the Schwäbische Alb and the Hainich-Dün (mixture of new and resident species).

### Ethics approval

All data collection complied with relevant institutional, national, and international guidelines and legislation. Field work permits (including allowances for the collection of plant material) were issued by the responsible state environmental offices of Baden-Württemberg, Thüringen, and Brandenburg (according to Sect. 72 BbgNatSchG). All plant species were identified in the field by Karl Andraczek, Fons van der Plas, Judith Hinderling, Alexandra Weigelt, Esther Rauwolf and Cristobal Cantuarias; and no voucher species were collected (only plant material to quantify actual standing biomass was collected, see Methods).

### Supplementary Information

Supplementary Information 1.

Supplementary Information 2.

Supplementary Information 3.

## Data Availability

Data and Code are available in the BExIS database of the Biodiversity Exploratories program https://www.bexis.uni-jena.de/ddm/data/Showdata/31215 (ID 31,215). The raw dataset with the ID 24,766 is available in the BExIS database under the URL https://www.bexis.uni-jena.de/ddm/publicsearch/index. Raw data sets with the ID 31,203, 31,204, 31,205 will be publicly available under the URL https://www.bexis.uni-jena.de/ddm/publicsearch/index from December 2023 on. The Data set with the ID 31,180 is publicly available under the URL https://www.bexis.uni-jena.de/ddm/publicsearch/index from December 2022 on. Until then, data is available upon request (bexis@uni-jena.de).
