# Supplementary material for: Relationships between species richness and biomass production are context dependent in grasslands differing in land-use and seed addition
Source: Sci Rep. 2023 Nov 11;13:19663. doi: 10.1038/s41598-023-47020-z (PMC10640580; doi:10.1038/s41598-023-47020-z)
Supplement: Supplementary file 2 — Supplementary Information 2. [file 41598_2023_47020_MOESM2_ESM.docx]

## Electronic Supplemental Material (ESM)

## Supplementary methods

**Phytometric Assay**

Soil fertility is often characterized by indicators of soil fertility, such as soil pH, soil texture, soil fragmentation or various soil nutrient concentrations (Grace and Bollen 2008). However, these measures may not fully represent the complexity of soil fertility, if the aim is to capture the suitability of soil for plant growth. We performed a phytometric assessment of potential productivity using biomass production in a controlled setting as a proxy for soil fertility to represent a more holistic view (Wheeler et al. 1992; Axmanová et al. 2012). As phytometer species we selected the herb species most frequently observed across all present land-use plots, i.e. *Taraxacum officinale* Kirschner H. Øllg. & Štěpánek*.* Seeds were purchased from a seed supplier (*Templiner Kräutergarten*, Templin, Germany). In spring 2021, we collected two soil cores (Ø 60 mm, 40.5 mm depth) from all subplots (544 samples in total). Samples were stored at 8°C until they were dried at 80°C for 48 hours. Then, dried soil samples were transferred into quick pots (75×70 mm, 230 cm^3^ per pot) in a randomized design with two replicates per plot (in total 544 pots), and cultivated in a greenhouse (Botanical Garden Leipzig) for 4 weeks from June to July 2021. After transferring the soil into the pots, all soil samples within the same quick-pot plate were irrigated from below with ~1500 ml for two days in a row, to avoid the impact of rapid changes in the microbial community after the first irrigation (Fierer and Schimel 2003). We shifted all quick-pot plates every two days, to randomize light conditions. All seeds of the phytometer species were pre-germinated in a growth chamber for 7 days at 20°C under light/dark cycle (16h/8h). After pre-germination, one seedling of *Taraxacum officinale* was transferred into each pot. To prevent that seedlings dried out during the early phase of the experiment we covered all pots from above in the first week of the experiment. During the experiment, each quick-pot plate with 24 pots per plate (23 quick-pot plates in total, for 544 soil samples) were watered from below every two days. All other plants germinating in the pots were weeded out biweekly. After four weeks, we harvested all aboveground plant biomass by clipping it to ground level. Harvested biomass was dried at 80°C for 48 hours and weighed.

## Supplementary figures and tables


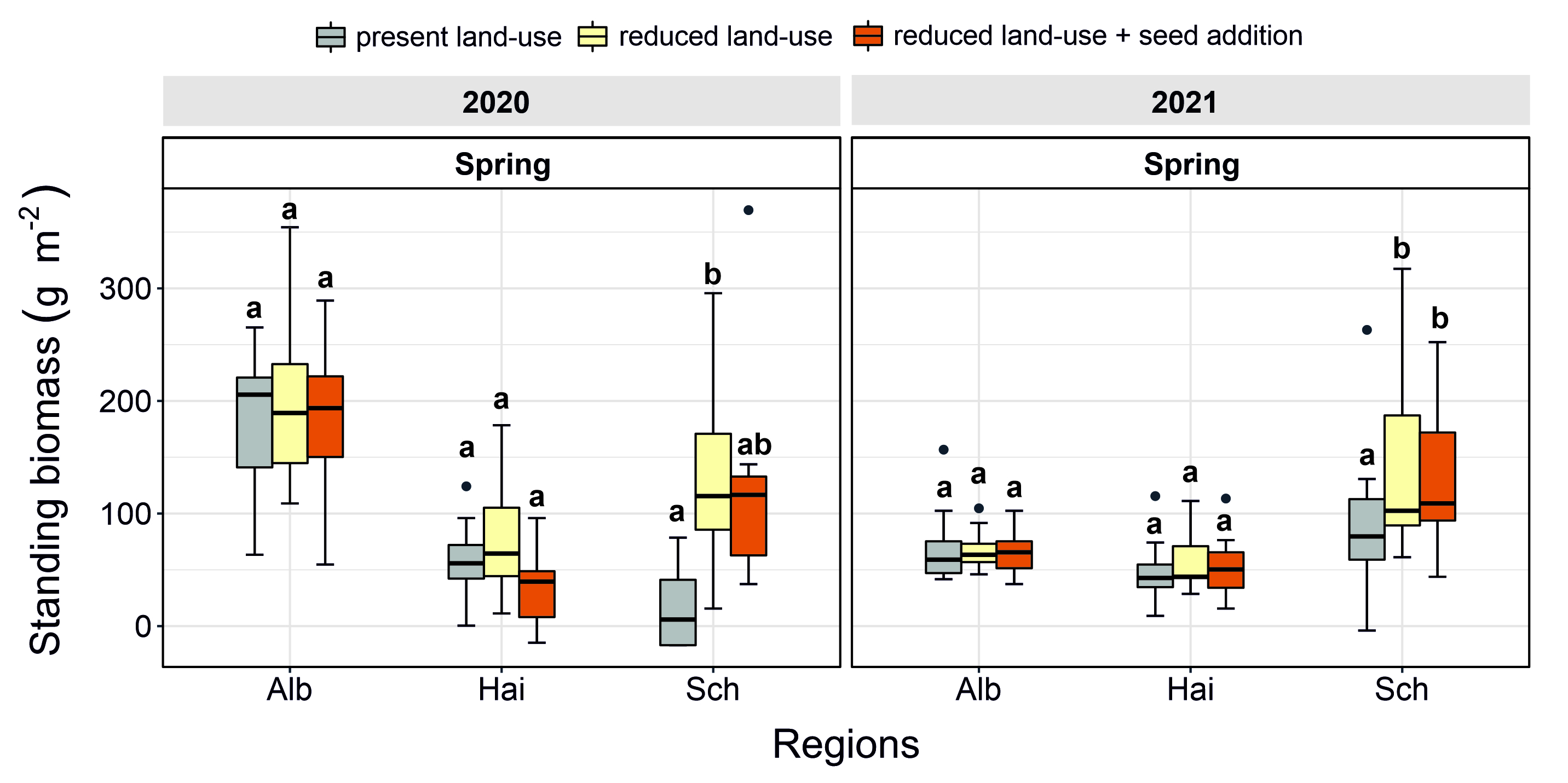


**Figure S1:** Response of standing biomass (g m^-2^) to land-use intensity in the subset of present land-use plots (plots located together in a field with a reduced land-use treatment), reduced land-use and reduced land-use + seed addition, in three regions (Alb: Schwäbische Alb; Sch: Schorfheide-Chorin; Hai: Hainich-Dün), as well as for spring in both 2020 and 2021. Present land-use: grey; reduced land-use: yellow; reduced land-use + seed addition: red. Bars sharing a letter (a,b) do not differ significantly (P<0.05, suppl. Table S12).


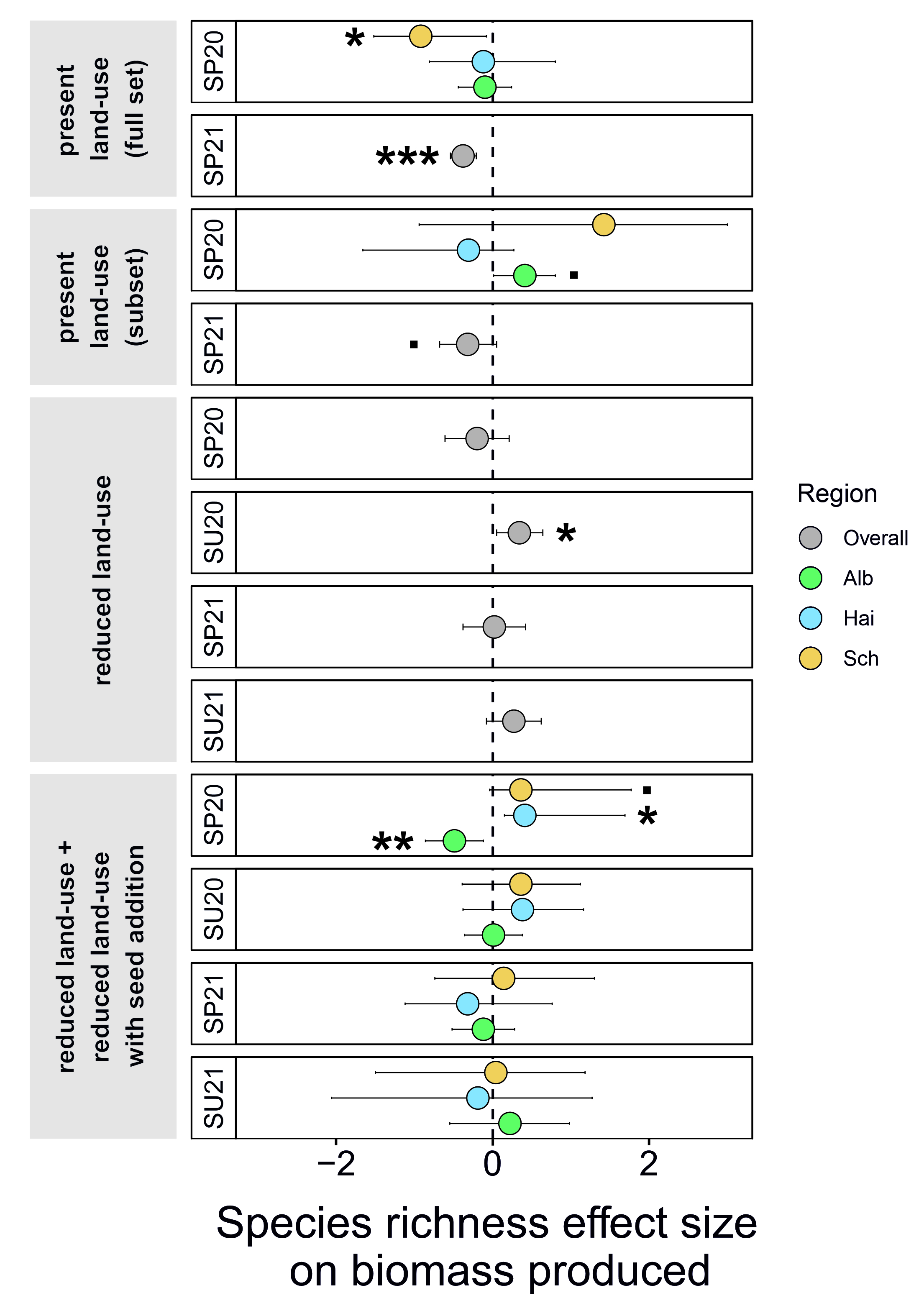


**Figure S2:** Relationships between species richness and biomass produced among different treatments and regions (Alb: Schwäbische Alb; Hai: Hainich-Dün; Sch: Schorfheide-Chorin) and contexts. Richness effects indicates the standardized effect of richness on biomass produced. present land-use (full set: full gradient of present land-use, subset: present land-use plots located together in a field with a reduced land-use treatment); reduced land-use, reduced land-use + seed addition. Effect sizes are colour coded for all three regions (Alb - Schwäbische Alb: green; Hai - Hainich-Dün: blue; Sch - Schorfheide-Chorin: yellow), if interaction between species richness and region was observed. When species richness effect did not differ between regions, effect size is colour coded in grey. Error bars indicating confidence intervals of each effect size. SU20: summer 2020, SP21: spring 2021, SU21: summer 2021. Significant effects are indicated following: 0 ‘***’ 0.001 ‘**’ 0.01 ‘*’ 0.05 ‘.’ 0.1 ‘ ’ 1 (for further information see suppl. Table S2-S4). Although some of the most parsimonious models include a squared term of species richness (always non-significant), we show only model without squared term to guarantee comparability between models.


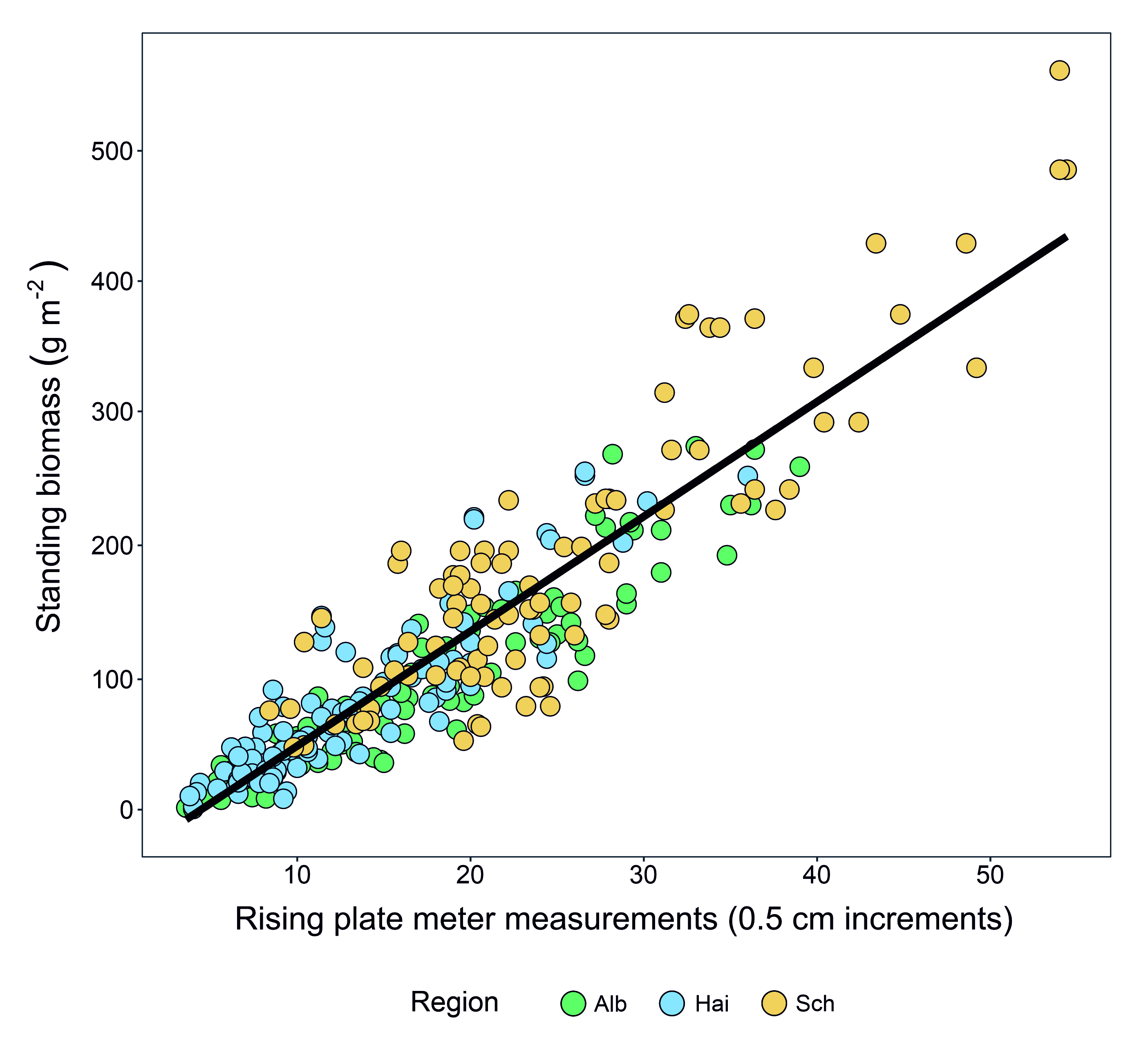


**Figure S3:** Calibration regression showing calibration data of rising-plate meter measurements (averaged per subplot) and standing biomass measurements from the same subplot (2 x 150 calibration subplots in all present land-use plots, 1×1 m each) from all three regions (Alb: Schwäbische Alb; Sch: Schorfheide-Chorin; Hai: Hainich-Dün) collected in spring 2021. Calibration regression was used to convert rising-plate meter measurements to standing biomass (in g m^-2^) following: Standing biomass = -38.6585 + 8.6840 * Biomass_PlateMeter. For further information see suppl. Table S13.

**Table S1:** Linear mixed effect model showing the effect of land-use reduction treatment (red. LU) and reduced land-use + seed addition treatment (red. LU + S) on species richness in comparison with the subset of present land-use subplots (subplots located together in a field with a reduced land-use treatment) for each regions (Alb: Schwäbische Alb; Sch: Schorfheide-Chorin; Hai: Hainich-Dün), as well as for different years and seasons.

|  | **Variable** | **Estimate** | **Std. Error** | **df** | **t** | **P** |
| --- | --- | --- | --- | --- | --- | --- |
| Spring 2020 | Intercept (Alb) | 17.18 | 0.86 | 70.51 | 19.89 | < 0.01 |
|  | Hai | -3.84 | 1.28 | 68.59 | -3.00 | < 0.01 |
|  | Sch | -3.28 | 1.62 | 102.68 | -2.03 | 0.05 |
|  | red. LU | -0.18 | 0.80 | 66.32 | -0.22 | 0.83 |
|  | red. LU + S | 0.09 | 0.80 | 66.32 | 0.11 | 0.91 |
|  | Hai: red. LU | -1.57 | 1.19 | 66.01 | -1.32 | 0.19 |
|  | Sch: red. LU | -2.39 | 1.56 | 72.02 | -1.54 | 0.13 |
|  | Hai: red. LU + S | -0.76 | 1.19 | 66.01 | -0.64 | 0.53 |
|  | Sch: red. LU + S | -0.99 | 1.56 | 72.02 | -0.64 | 0.53 |
| Summer 2020 | Intercept (Alb) | 13.58 | 0.93 | 81.17 | 14.66 | < 0.01 |
|  | Hai | -0.45 | 0.86 | 84.09 | -0.52 | 0.60 |
|  | Sch | 0.08 | 0.86 | 84.09 | 0.10 | 0.92 |
|  | red. LU | -1.33 | 1.28 | 79.24 | -1.04 | 0.30 |
|  | red. LU + S | -2.45 | 1.30 | 79.17 | -1.89 | 0.06 |
|  | Hai: red. LU | -0.88 | 1.20 | 84.53 | -0.73 | 0.47 |
|  | Sch: red. LU | 2.39 | 1.20 | 84.53 | 2.00 | 0.05 |
|  | Hai: red. LU + S | -0.55 | 1.20 | 83.74 | -0.46 | 0.65 |
|  | Sch: red. LU + S | 3.85 | 1.20 | 83.74 | 3.20 | < 0.01 |
| Spring 2021 | Intercept (Alb) | 16.87 | 0.97 | 75.91 | 17.34 | < 0.01 |
|  | Hai | 0.13 | 0.92 | 79.41 | 0.14 | 0.89 |
|  | Sch | 2.87 | 0.92 | 79.41 | 3.10 | < 0.01 |
|  | red. LU | -1.74 | 1.35 | 75.91 | -1.29 | 0.20 |
|  | red. LU + S | -2.47 | 1.45 | 78.41 | -1.71 | 0.09 |
|  | Hai: red. LU | -1.43 | 1.30 | 80.20 | -1.10 | 0.28 |
|  | Sch: red. LU | 0.11 | 1.30 | 80.20 | 0.08 | 0.93 |
|  | Hai: red. LU + S | 0.16 | 1.38 | 79.86 | 0.12 | 0.91 |
|  | Sch: red. LU + S | 1.20 | 1.38 | 79.86 | 0.87 | 0.39 |
| Summer 2021 | Intercept (Alb) | 16.47 | 1.04 | 92.61 | 15.90 | < 0.01 |
|  | Hai | 0.13 | 1.10 | 84.44 | 0.12 | 0.90 |
|  | Sch | 2.33 | 1.10 | 84.44 | 2.12 | 0.04 |
|  | red. LU | -2.15 | 1.44 | 92.61 | -1.49 | 0.14 |
|  | red. LU + S | -3.67 | 1.47 | 92.61 | -2.50 | 0.01 |
|  | Hai: red. LU | 0.02 | 1.55 | 85.38 | 0.01 | 0.99 |
|  | Sch: red. LU | 4.15 | 1.55 | 85.38 | 2.68 | 0.01 |
|  | Hai: red. LU + S | -0.07 | 1.56 | 84.44 | -0.04 | 0.97 |
|  | Sch: red. LU + S | 2.40 | 1.56 | 84.44 | 1.54 | 0.13 |

**Table S2:** Linear model showing the effect of species richness and potential covarying factors in the full set and the subset of the present land-use treatment (full set: full gradient of present land-use; subset: reduced gradient of present land-use, representing subplots located together in a field with a reduced land-use treatment) on biomass produced (g m^-2^) for each regions (Alb: Schwäbische Alb; Sch: Schorfheide-Chorin; Hai: Hainich-Dün), as well as for different years and seasons.

|  |  | **Variable** | **Estimate** | **Std. Error** | **t** | **P** |
| --- | --- | --- | --- | --- | --- | --- |
| Full set | Spring 2020 | (Intercept) Alb | 123.67 | 80.47 | 1.54 | 0.13 |
|  |  | Hai | -85.90 | 101.35 | -0.85 | 0.40 |
|  |  | Sch | 156.16 | 89.90 | 1.74 | 0.09 |
|  |  | Species richness | -2.42 | 3.35 | -0.72 | 0.47 |
|  |  | Hai : Species richness | -0.34 | 6.38 | -0.05 | 0.96 |
|  |  | Sch : Species richness | -15.57 | 6.89 | -2.26 | 0.03 |
|  |  | Soil moisture | 1.52 | 1.23 | 1.24 | 0.22 |
|  |  | Potential productivity | 532.45 | 621.59 | 0.86 | 0.39 |
|  | Spring 2021 | (Intercept) | -313.25 | 107.39 | -2.92 | < 0.01 |
|  |  | Species richness | -4.06 | 0.84 | -4.81 | < 0.01 |
|  |  | Soil moisture | 0.71 | 0.64 | 1.11 | 0.27 |
|  |  | Potential productivity | 87.05 | 267.66 | 0.33 | 0.75 |
|  |  | Sampling date | 3.33 | 0.79 | 4.20 | < 0.01 |
| Subset | Spring 2020 | (Intercept) Alb | 314.23 | 135.05 | 2.33 | 0.03 |
|  |  | Hai | -117.41 | 23.30 | -5.04 | < 0.01 |
|  |  | Sch | -153.58 | 34.55 | -4.45 | < 0.01 |
|  |  | Species richness | -23.93 | 16.38 | -1.46 | 0.16 |
|  |  | Species richness (quadratic) | 0.89 | 0.50 | 1.76 | 0.09 |
|  |  | Potential productivity | 408.97 | 606.30 | 0.68 | 0.51 |
|  | Spring 2021 | (Intercept) | -196.62 | 144.48 | -1.36 | 0.19 |
|  |  | Species richness | -3.13 | 1.66 | -1.89 | 0.07 |
|  |  | Soil moisture | 1.27 | 0.90 | 1.41 | 0.17 |
|  |  | Potential productivity | 308.39 | 332.78 | 0.93 | 0.36 |
|  |  | Sampling date | 2.03 | 1.07 | 1.91 | 0.07 |

**Table S3:** Linear model showing the effect of species richness and potential covarying factors when reducing land-use on biomass produced (g m^-2^) for each regions (Alb: Schwäbische Alb; Sch: Schorfheide-Chorin; Hai: Hainich-Dün), as well as years and seasons.

|  | **Variable** | **Estimate** | **Std. Error** | **t** | **P** |
| --- | --- | --- | --- | --- | --- |
| Spring 2020 | (Intercept) Alb | 327.89 | 120.89 | 2.71 | 0.01 |
|  | Hai | -150.66 | 37.47 | -4.02 | < 0.01 |
|  | Sch | -126.79 | 49.47 | -2.56 | 0.02 |
|  | Species richness | -4.40 | 3.96 | -1.11 | 0.28 |
|  | Potential productivity | 116.90 | 678.29 | 0.17 | 0.86 |
|  | Soil moisture | -1.67 | 2.05 | -0.82 | 0.42 |
| Summer 2020 | (Intercept) | -1749.00 | 451.00 | -3.88 | < 0.01 |
|  | Species richness | -17.13 | 21.02 | -0.82 | 0.42 |
|  | Species richness (quadratic) | 1.07 | 0.87 | 1.23 | 0.23 |
|  | Potential productivity | 791.20 | 768.70 | 1.03 | 0.31 |
|  | Sampling date | 8.78 | 1.92 | 4.58 | < 0.01 |
|  | Soil moisture | 0.05 | 1.56 | 0.03 | 0.97 |
|  | LUI | -28.37 | 25.09 | -1.13 | 0.27 |
| Spring 2021 | (Intercept) Alb | 64.73 | 113.65 | 0.57 | 0.57 |
|  | Hai | -1.96 | 22.88 | -0.09 | 0.93 |
|  | Sch | 67.01 | 29.15 | 2.30 | 0.03 |
|  | Species richness | 0.03 | 2.66 | 0.01 | 0.99 |
|  | Soil moisture | -0.34 | 1.84 | -0.19 | 0.85 |
|  | Potential productivity | 503.00 | 722.29 | 0.70 | 0.49 |
| Summer 2021 | (Intercept) | -658.40 | 736.36 | -0.89 | 0.38 |
|  | Species richness | 7.32 | 4.98 | 1.47 | 0.15 |
|  | Soil moisture | -3.56 | 2.21 | -1.61 | 0.12 |
|  | Potential productivity | -2776.90 | 1297.66 | -2.14 | 0.04 |
|  | Sampling date | 4.24 | 3.20 | 1.32 | 0.20 |

**Table S4:** Linear mixed effect model showing the effect of species richness and potential covarying factors when reducing land-use and reducing land-use + seed addition on biomass produced (g m^-2^) for each regions (Alb: Schwäbische Alb; Sch: Schorfheide-Chorin; Hai: Hainich-Dün), as well as years and seasons.

|  | **Variable** | **Estimate** | **Std. Error** | **df** | **t** | **P** |
| --- | --- | --- | --- | --- | --- | --- |
| Spring 2020 | (Intercept) Alb | 378.50 | 96.44 | 41.14 | 3.93 | 0.00 |
|  | Species richness | -2.73 | 2.35 | 62.03 | -1.17 | 0.25 |
|  | Hai: Species richness | -166.10 | 26.15 | 41.03 | -6.35 | 0.00 |
|  | Sch: Species richness | -143.37 | 36.92 | 38.19 | -3.88 | 0.00 |
|  | Potential productivity | 168.44 | 405.47 | 62.38 | 0.42 | 0.68 |
|  | LUI | -22.17 | 19.61 | 34.35 | -1.13 | 0.27 |
|  | Soil moisture | -2.64 | 1.59 | 32.97 | -1.66 | 0.11 |
| Summer 2020 | (Intercept) Alb | 35.23 | 77.83 | 47.26 | 0.45 | 0.65 |
|  | Hai | 62.97 | 79.51 | 62.91 | 0.79 | 0.43 |
|  | Sch | 27.01 | 75.31 | 60.45 | 0.36 | 0.72 |
|  | Species richness | -0.15 | 4.69 | 57.80 | -0.03 | 0.97 |
|  | Hai: Species richness | 5.47 | 5.85 | 62.29 | 0.94 | 0.35 |
|  | Sch: Species richness | 4.74 | 5.27 | 62.16 | 0.90 | 0.37 |
|  | Potential productivity | 45.86 | 441.35 | 54.22 | 0.10 | 0.92 |
|  | LUI | -11.54 | 24.01 | 35.86 | -0.48 | 0.63 |
| Spring 2021 | (Intercept) Alb | 137.60 | 91.61 | 34.44 | 1.50 | 0.14 |
|  | Hai | 4.85 | 51.10 | 47.02 | 0.10 | 0.93 |
|  | Sch | 29.63 | 57.72 | 50.67 | 0.51 | 0.61 |
|  | Species richness | -1.74 | 2.54 | 42.77 | -0.69 | 0.50 |
|  | Hai : Species richness | -1.19 | 2.84 | 39.86 | -0.42 | 0.68 |
|  | Sch : Species richness | 1.59 | 3.17 | 41.29 | 0.50 | 0.62 |
|  | Soil moisture | -0.95 | 1.44 | 29.40 | -0.66 | 0.52 |
|  | Potential productivity | 269.16 | 244.73 | 38.59 | 1.10 | 0.28 |
|  | LUI | -5.08 | 16.10 | 25.61 | -0.32 | 0.76 |
| Summer 2021 | (Intercept) Alb | 223.16 | 168.09 | 40.73 | 1.33 | 0.19 |
|  | Hai | 113.37 | 142.55 | 49.02 | 0.80 | 0.43 |
|  | Sch | 35.26 | 153.92 | 49.34 | 0.23 | 0.82 |
|  | Species richness | 3.86 | 7.61 | 48.40 | 0.51 | 0.62 |
|  | Hai : Species richness | -3.90 | 8.01 | 50.34 | -0.49 | 0.63 |
|  | Sch : Species richness | -2.39 | 9.07 | 52.66 | -0.26 | 0.79 |
|  | Soil moisture | -3.13 | 2.68 | 30.16 | -1.17 | 0.25 |
|  | Potential productivity | -927.24 | 690.35 | 48.77 | -1.34 | 0.19 |

**Table S5:** Linear model showing the effect of Delta species richness (between reduced land-use + seed addition and reduced land-use subplots) on Delta biomass produced (g m^-2^, between reduced land-use + seed addition and reduced land-use subplots), for each year and season.

|  | **Variable** | **Estimate** | **Std. Error** | **t** | **P** |
| --- | --- | --- | --- | --- | --- |
| Spring 2020 | (Intercept) | -20.80 | 9.14 | -2.28 | 0.03 |
|  | Delta species richness | 1.42 | 3.21 | 0.44 | 0.66 |
| Summer 2020 | (Intercept) | 1.17 | 11.90 | 0.10 | 0.92 |
|  | Delta species richness | 2.68 | 2.53 | 1.06 | 0.30 |
| Spring 2021 | (Intercept) | 1.52 | 6.42 | 0.24 | 0.81 |
|  | Delta species richness | -0.81 | 2.88 | -0.28 | 0.78 |
|  | Delta species richness (quadratic) | -0.21 | 0.40 | -0.54 | 0.60 |
| Summer 2021 | (Intercept) | -23.62 | 16.27 | -1.45 | 0.15 |
|  | Delta species richness | 2.71 | 2.68 | 1.01 | 0.32 |

**Table S6:** Linear mixed effect model showing the effect of reduced land-use (red. LU) and reduced land-use + seed addition (red. LU + S) on standing biomass (g m^-2^) in comparison with the subset of present land-use subplots (subplots located together in a field with a reduced land-use treatment) for each regions (Alb: Schwäbische Alb; Sch: Schorfheide-Chorin; Hai: Hainich-Dün), as well as for different years and seasons.

|  | **Variable** | **Estimate** | **Std. Error** | **df** | **t** | **P** |
| --- | --- | --- | --- | --- | --- | --- |
| Spring 2020 | Intercept (Alb) | 188.99 | 16.86 | 75.69 | 11.21 | < 0.01 |
|  | Hai | -131.22 | 24.96 | 73.60 | -5.26 | < 0.01 |
|  | Sch | -134.47 | 32.85 | 102.60 | -4.09 | < 0.01 |
|  | red. LU | 6.39 | 17.34 | 61.88 | 0.37 | 0.71 |
|  | red. LU + S | -1.14 | 17.34 | 61.88 | -0.07 | 0.95 |
|  | Hai: red. LU | 8.08 | 25.69 | 61.47 | 0.32 | 0.75 |
|  | Sch: red. LU | 70.19 | 33.43 | 69.50 | 2.10 | 0.04 |
|  | Hai: red. LU + S | -21.48 | 25.69 | 61.47 | -0.84 | 0.41 |
|  | Sch: red. LU + S | 62.56 | 33.43 | 69.50 | 1.87 | 0.07 |
| Spring 2021 | Intercept (Alb) | 67.58 | 11.57 | 72.16 | 5.84 | < 0.01 |
|  | Hai | -20.89 | 16.11 | 72.16 | -1.30 | 0.20 |
|  | Sch | 22.65 | 17.19 | 74.52 | 1.32 | 0.19 |
|  | red. LU | 0.29 | 10.49 | 79.61 | 0.03 | 0.98 |
|  | red. LU + S | -2.89 | 10.49 | 79.61 | -0.28 | 0.78 |
|  | Hai: red. LU | 12.50 | 14.79 | 80.36 | 0.85 | 0.40 |
|  | Sch: red. LU | 8.16 | 14.79 | 80.36 | 0.55 | 0.58 |
|  | Hai: red. LU + S | 54.70 | 15.63 | 80.02 | 3.50 | < 0.01 |
|  | Sch: red. LU + S | 50.03 | 15.63 | 80.02 | 3.20 | < 0.01 |

**Table S7:** Linear mixed effect model showing the effect of reduced land-use (red. LU) and reduced land-use + seed addition (red. LU + S) on Shannon Diversity in comparison with the subset of present land-use subplots (subplots located together in a field with a reduced land-use treatment) for each regions (Alb: Schwäbische Alb; Sch: Schorfheide-Chorin; Hai: Hainich-Dün), as well as for different years and seasons.

|  | **Variable** | **Estimate** | **Std. Error** | **df** | **t** | **P** |
| --- | --- | --- | --- | --- | --- | --- |
| Spring 2020 | Intercept | 2.30 | 0.07 | 35.64 | 30.90 | < 0.01 |
|  | Hai | -0.78 | 0.11 | 35.38 | -6.98 | < 0.01 |
|  | Sch | -0.53 | 0.11 | 38.42 | -4.97 | < 0.01 |
| Summer 2020 | Intercept | 1.86 | 0.05 | 45.19 | 40.26 | < 0.01 |
| Spring 2021 | Intercept | 2.03 | 0.05 | 42.93 | 37.09 | < 0.01 |
| Summer 2021 | Intercept | 2.01 | 0.05 | 82.67 | 39.35 | < 0.01 |
|  | red. LU | 0.05 | 0.05 | 87.29 | 0.95 | 0.34 |
|  | red. LU + S | 0.20 | 0.05 | 87.29 | 4.04 | < 0.01 |

**Table S8:** Linear mixed effect model showing the effect of reduced land-use + seed addition treatment (red. LU + S) and reduced land-use + soil scarifying (red. LU + SC) on species richness in comparison to the reduced land-use treatment, for each region (Alb: Schwäbische Alb; Sch: Schorfheide-Chorin; Hai: Hainich-Dün), for spring 2020.

|  | **Variable** | **Estimate** | **Std. Error** | **df** | **t** | **P** |
| --- | --- | --- | --- | --- | --- | --- |
| Spring 2020 | Intercept (Alb) | 17.91 | 1.15 | 16.07 | 15.57 | < 0.01 |
|  | red. LU + SC | 0.67 | 0.75 | 28.00 | 0.89 | 0.38 |
|  | red. LU + S | 0.27 | 0.75 | 28.00 | 0.36 | 0.72 |
|  | Hai | -7.96 | 1.58 | 12.00 | -5.03 | < 0.01 |
|  | Sch | -4.97 | 1.69 | 12.00 | -2.95 | 0.01 |

**Table S9:** Species richness, standing biomass and Shannon Diversity across the subset of present land-use subplots (subplots located together in a field with a reduced land-use treatment), the reduced land-use treatment, and the reduced land-use + seed addition treatment, for each region (Alb: Schwäbische Alb; Sch: Schorfheide-Chorin; Hai: Hainich-Dün), as well as for different years and seasons.

|  | **Treatment** | **Region** | **Spring**  **2020** | **Summer**  **2020** | **Spring**  **2021** | **Summer**  **2021** |
| --- | --- | --- | --- | --- | --- | --- |
| Species  richness | Pres. LU | Alb | 17.29 (n=14) | 13.79 (n=14) | 16.87 (n=15) | 16.47 (n=15) |
|  | Pres. LU | Hai | 13.33 (n=12) | 12.25 (n=16) | 15.12 (n=16) | 14.31 (n=16) |
|  | Pres. LU | Sch | 14.50 (n=4) | 11.13 (n=15) | 14.17 (n=12) | 12.80 (n=15) |
|  | Red. LU | Alb | 17.00 (n=15) | 13.13 (n=15) | 17.00 (n=15) | 16.60 (n=15) |
|  | Red. LU | Hai | 11.58 (n=12) | 10.93 (n=15) | 13.80 (n=15) | 14.53 (n=15) |
|  | Red. LU | Sch | 11.33 (n=15) | 10.13 (n=15) | 14.69 (n=13) | 12.87 (n=15) |
|  | Red. LU + S | Alb | 17.27 (n=15) | 13.67 (n=15) | 19.73 (n=15) | 18.80 (n=15) |
|  | Red. LU + S | Hai | 12.67 (n=12) | 14.73 (n=15) | 18.07 (n=15) | 20.87 (n=15) |
|  | Red. LU + S | Sch | 13.00 (n=15) | 15.07 (n=15) | 18.46 (n=13) | 17.53 (n=15) |
| Shannon  Diversity | Pres. LU | Alb | 2.32 (n=14) | 1.85 (n=14) | 2.16 (n=15) | 2.13 (n=15) |
|  | Pres. LU | Hai | 1.59 (n=12) | 1.82 (n=16) | 1.83 (n=16) | 1.96 (n=16) |
|  | Pres. LU | Sch | 2.15 (n=4) | 1.75 (n=15) | 1.90 (n=12) | 1.94 (n=15) |
|  | Red. LU | Alb | 2.31 (n=15) | 1.87 (n=15) | 2.17 (n=15) | 2.14 (n=15) |
|  | Red. LU | Hai | 1.43 (n=12) | 1.73 (n=15) | 1.92 (n=15) | 2.06 (n=15) |
|  | Red. LU | Sch | 1.71 (n=15) | 1.80 (n=15) | 1.88 (n=13) | 1.99 (n=15) |
|  | Red. LU + S | Alb | 2.26 (n=15) | 1.97 (n=15) | 2.24 (n=15) | 2.21 (n=15) |
|  | Red. LU + S | Hai | 1.54 (n=12) | 1.92 (n=15) | 2.03 (n=15) | 2.28 (n=15) |
|  | Red. LU + S | Sch | 1.76 (n=15) | 2.01 (n=15) | 2.07 (n=13) | 2.17 (n=15) |
| Standing  biomass | Pres. LU | Alb | 183.09 (n=14) | 62.91 (n=14) | 67.58 (n=15) | 163.53 (n=15) |
|  | Pres. LU | Hai | 57.77 (n=12) | 79.52 (n=16) | 46.69 (n=16) | 176.27 (n=16) |
|  | Pres. LU | Sch | 18.33 (n=4) | 83.21 (n=15) | 90.88 (n=12) | 181.91 (n=15) |
|  | Red. LU | Alb | 195.37 (n=15) | 210.14 (n=15) | 67.86 (n=15) | 240.24 (n=15) |
|  | Red. LU | Hai | 72.24 (n=12) | 194.65 (n=15) | 59.76 (n=15) | 300.6 (n=15) |
|  | Red. LU | Sch | 130.52 (n=14) | 211.3 (n=15) | 145.21 (n=13) | 320.86 (n=15) |
|  | Red. LU + S | Alb | 187.85 (n=15) | 189.15 (n=15) | 64.68 (n=15) | 226.35 (n=15) |
|  | Red. LU + S | Hai | 35.16 (n=12) | 179.6 (n=15) | 52.23 (n=15) | 265.28 (n=15) |
|  | Red. LU + S | Sch | 117.19 (n=14) | 215.64 (n=15) | 137.36 (n=13) | 319.7 (n=15) |
|  | Red. LU + S | Sch | 117.19 (n=14) | 215.64 (n=15) | 137.36 (n=13) | 319.7 (n=15) |

**Table S10:** Results from TuckeyHSD test showing Pairwise comparisons of the species richness in the subset of present land-use subplots (pres. LU, subplots located together in a field with a reduced land-use treatment), the reduced land-use treatment (red. LU), and the reduced land-use + seed addition treatment (red. LU + S), for each region (Alb: Schwäbische Alb; Sch: Schorfheide-Chorin; Hai: Hainich-Dün), as well as for different years and seasons. Significant (< 0.05) contrasts are written in bold.

|  | **Variable** | **contrast** | **estimate** | **SE** | **df** | **t** | **P** |
| --- | --- | --- | --- | --- | --- | --- | --- |
| Spring 2020 | Alb | pres. LU - red. LU | 0.18 | 0.80 | 66.70 | 0.22 | 0.97 |
|  |  | pres. LU - red. LU + S | -0.09 | 0.80 | 66.70 | -0.11 | 0.99 |
|  |  | red. LU - red. LU + S | -0.27 | 0.79 | 66.10 | -0.34 | 0.94 |
|  | Hai | pres. LU - red. LU | 1.75 | 0.88 | 66.10 | 1.99 | 0.12 |
|  |  | pres. LU - red. LU + S | 0.67 | 0.88 | 66.10 | 0.76 | 0.73 |
|  |  | red. LU - red. LU + S | -1.08 | 0.88 | 66.10 | -1.23 | 0.44 |
|  | Sch | pres. LU - red. LU | 2.56 | 1.34 | 74.50 | 1.92 | 0.14 |
|  |  | pres. LU - red. LU + S | 0.90 | 1.34 | 74.50 | 0.67 | 0.78 |
|  |  | red. LU - red. LU + S | -1.67 | 0.79 | 66.10 | -2.12 | 0.09 |
| Summer 2020 | Alb | pres. LU - red. LU | 0.45 | 0.86 | 83.80 | 0.52 | 0.86 |
|  |  | pres. LU - red. LU + S | -0.08 | 0.86 | 83.80 | -0.10 | 0.99 |
|  |  | red. LU - red. LU + S | -0.53 | 0.84 | 83.10 | -0.64 | 0.80 |
|  | Hai | pres. LU - red. LU | 1.33 | 0.83 | 84.70 | 1.59 | 0.26 |
|  |  | **pres. LU - red. LU + S** | -2.47 | 0.83 | 84.70 | -2.97 | **0.01** |
|  |  | **red. LU - red. LU + S** | -3.80 | 0.84 | 83.10 | -4.53 | **< 0.01** |
|  | Sch | pres. LU - red. LU | 1.00 | 0.84 | 83.10 | 1.19 | 0.46 |
|  |  | **pres. LU - red. LU + S** | -3.93 | 0.84 | 83.10 | -4.69 | **< 0.01** |
|  |  | **red. LU - red. LU + S** | -4.93 | 0.84 | 83.10 | -5.88 | **< 0.01** |
| Spring 2021 | Alb | pres. LU - red. LU | -0.13 | 0.92 | 79.10 | -0.14 | 0.99 |
|  |  | **pres. LU - red. LU + S** | -2.87 | 0.92 | 79.10 | -3.10 | **0.01** |
|  |  | **red. LU - red. LU + S** | -2.73 | 0.92 | 79.10 | -2.96 | **0.01** |
|  | Hai | pres. LU - red. LU | 1.29 | 0.92 | 80.70 | 1.41 | 0.34 |
|  |  | **pres. LU - red. LU + S** | -2.97 | 0.92 | 80.70 | -3.24 | **< 0.01** |
|  |  | **red. LU - red. LU + S** | -4.27 | 0.92 | 79.10 | -4.62 | **< 0.01** |
|  | Sch | pres. LU - red. LU | -0.29 | 1.02 | 79.90 | -0.29 | 0.96 |
|  |  | **pres. LU - red. LU + S** | -4.06 | 1.02 | 79.90 | -3.98 | **< 0.01** |
|  |  | **red. LU - red. LU + S** | -3.77 | 0.99 | 79.10 | -3.80 | **< 0.01** |
| Summer 2021 | Alb | pres. LU - red. LU | -0.13 | 1.10 | 84.10 | -0.12 | 0.99 |
|  |  | pres. LU - red. LU + S | -2.33 | 1.10 | 84.10 | -2.12 | 0.09 |
|  |  | red. LU - red. LU + S | -2.20 | 1.10 | 84.10 | -2.00 | 0.12 |
|  | Hai | pres. LU - red. LU | -0.15 | 1.09 | 86.00 | -0.14 | 0.99 |
|  |  | **pres. LU - red. LU + S** | -6.49 | 1.09 | 86.00 | -5.94 | **< 0.01** |
|  |  | **red. LU - red. LU + S** | -6.33 | 1.10 | 84.10 | -5.75 | **< 0.01** |
|  | Sch | pres. LU - red. LU | -0.07 | 1.10 | 84.10 | -0.06 | 0.99 |
|  |  | **pres. LU - red. LU + S** | -4.73 | 1.10 | 84.10 | -4.30 | **< 0.01** |
|  |  | **red. LU - red. LU + S** | -4.67 | 1.10 | 84.10 | -4.24 | **< 0.01** |

**Table S11:** Results from TuckeyHSD test showing Pairwise comparisons of the Shannon Diversity in the subset of present land-use subplots (pres. LU, subplots located together in a field with a reduced land-use treatment), the reduced land-use treatment (red. LU), and the reduced land-use + seed addition treatment (red. LU + S), Summer 2021 (previous seasons and year not shown as treatment was not part of the most parsimonious models). Significant (< 0.05) contrasts are written in bold.

|  | **contrast** | **estimate** | **SE** | **df** | **t** | **P** |
| --- | --- | --- | --- | --- | --- | --- |
| Summer 2021 | pres. LU - red. LU | -0.05 | 0.05 | 88.70 | -0.95 | 0.61 |
|  | **pres. LU - red. LU + S** | -0.20 | 0.05 | 88.70 | -4.03 | **< 0.01** |
|  | **red. LU - red. LU + S** | -0.15 | 0.05 | 88.10 | -3.07 | **0.01** |

**Table S12:** Results from TuckeyHSD test showing Pairwise comparisons of the Standing biomass (g m^-2^) in the subset of present land-use subplots (pres. LU, subplots located together in a field with a reduced land-use treatment), the reduced land-use treatment (red. LU), and the reduced land-use + seed addition treatment (red. LU + S), for different years and seasons. Significant (< 0.05) contrasts are written in bold.

|  | **Variable** | **contrast** | **estimate** | **SE** | **df** | **t** | **P** |
| --- | --- | --- | --- | --- | --- | --- | --- |
| Spring 2020 | Alb | pres. LU - red. LU | -6.39 | 17.3 | 65.1 | -0.37 | 0.93 |
|  |  | pres. LU - red. LU + S | 1.14 | 17.3 | 65.1 | 0.07 | 1.00 |
|  |  | red. LU - red. LU + S | 7.53 | 16.9 | 64.3 | 0.44 | 0.90 |
|  | Hai | pres. LU - red. LU | -14.47 | 18.9 | 64.3 | -0.76 | 0.73 |
|  |  | pres. LU - red. LU + S | 22.61 | 18.9 | 64.3 | 1.19 | 0.46 |
|  |  | red. LU - red. LU + S | 37.09 | 18.9 | 64.3 | 1.96 | 0.13 |
|  | Sch | **pres. LU - red. LU** | -76.58 | 28.8 | 75.2 | -2.66 | **0.03** |
|  |  | pres. LU - red. LU + S | -61.42 | 28.8 | 75.2 | -2.14 | 0.09 |
|  |  | red. LU - red. LU + S | 15.16 | 17.9 | 67.8 | 0.85 | 0.67 |
| Spring 2021 | Alb | pres. LU - red. LU | -0.289 | 10.5 | 79.1 | -0.03 | 1.00 |
|  |  | pres. LU - red. LU + S | 2.895 | 10.5 | 79.1 | 0.28 | 0.96 |
|  |  | red. LU - red. LU + S | 3.184 | 10.5 | 79.1 | 0.30 | 0.95 |
|  | Hai | pres. LU - red. LU | -12.789 | 10.4 | 80.6 | -1.23 | 0.44 |
|  |  | pres. LU - red. LU + S | -5.263 | 10.4 | 80.6 | -0.51 | 0.87 |
|  |  | red. LU - red. LU + S | 7.526 | 10.5 | 79.1 | 0.72 | 0.75 |
|  | Sch | **pres. LU - red. LU** | -54.986 | 11.6 | 79.8 | -4.74 | **< 0.01** |
|  |  | **pres. LU - red. LU + S** | -47.137 | 11.6 | 79.8 | -4.07 | **< 0.01** |
|  |  | red. LU - red. LU + S | 7.849 | 11.3 | 79.1 | 0.70 | 0.77 |

**Table S13:** Calibration model (linear model) between standard biomass measurements (dry matter in g m^-2^) and rising plate meter measurement (1/2 cm increments), combined for all regions. Calibration was performed for spring 2021.

|  | **Estimate** | **Std. Error** | **t** | **P** |
| --- | --- | --- | --- | --- |
| (Intercept) | -38.66 | 4.70 | -8.23 | < 0.01 |
| Plate meter measurement | 8.68 | 0.23 | 37.94 | < 0.01 |

**Table S14:** Mean LUI components (grazing, mowing and fertilization) averaged across 2017 to 2019 for each region separately (Alb: Schwäbische Alb, Hai: Hainich-Dün, Sch: Schorfheide-Chorin).

| **Region** | **Grazing**  (Livestock units * d ha^-1^ year^-1^) | **Mowing**  (cuts year^-1^) | **Fertilization**  (kg N m^-3^ year^-1^) |
| --- | --- | --- | --- |
| Alb | 0.61 | 1.36 | 1.62 |
| Hai | 1.01 | 0.78 | 1.28 |
| Sch | 1.37 | 0.85 | 0.10 |

**Table S15:** Sensitivity analysis of linear model showing the effect of species richness and potential covarying factors when reducing land-use and reducing land-use + seed addition on biomass produced (g m^-2^) only for the Schorfheide-Chorin (only new seeds sown), as well as years and seasons.

|  | Variable | Estimate | Std. Error | t | P |
| --- | --- | --- | --- | --- | --- |
| Spring 2020 | (Intercept) | -150.60 | 114.09 | -1.32 | 0.21 |
|  | Species richness | 9.84 | 4.88 | 2.02 | 0.06 |
|  | Potential productivity | 952.20 | 768.48 | 1.24 | 0.23 |
|  | Soil moisture | 5.05 | 2.36 | 2.14 | 0.05 |
| Summer 2020 | (Intercept) | -4927.94 | 3238.00 | -1.52 | 0.15 |
|  | Richness | -9.16 | 17.90 | -0.51 | 0.62 |
|  | Richness (quadratic) | 0.47 | 0.64 | 0.74 | 0.48 |
|  | Potential productivity | -39.52 | 1021.22 | -0.04 | 0.97 |
|  | Sampling date | 23.10 | 14.62 | 1.58 | 0.14 |
|  | Soil moisture | 2.37 | 3.66 | 0.65 | 0.53 |
| Spring 2021 | (Intercept) | -7066.05 | 3471.33 | -2.04 | 0.07 |
|  | Species richness | 3.95 | 3.74 | 1.06 | 0.32 |
|  | Soil moisture | 0.62 | 2.79 | 0.22 | 0.83 |
|  | Potential productivity | 3221.58 | 1810.44 | 1.78 | 0.11 |
|  | Sampling date | 53.86 | 26.88 | 2.00 | 0.07 |
| Summer 2021 | (Intercept) | 728.28 | 281.76 | 2.59 | 0.03 |
|  | Species richness | -3.07 | 6.71 | -0.46 | 0.66 |
|  | Soil moisture | -10.34 | 5.81 | -1.78 | 0.11 |
|  | Potential productivity | -3289.67 | 2925.59 | -1.12 | 0.29 |
|  | LUI | -106.88 | 66.47 | -1.61 | 0.14 |

**Table S16:** Sensitivity analysis of linear mixed effect model showing the effect of species richness and potential covarying factors when reducing land-use and reducing land-use + seed addition on biomass produced (g m^-2^) only for the Schwäbische Alb and Hainich-Dün (mixture of resident and new seeds sown), as well as years and seasons.

|  | Variable | Estimate | Std. Error | df | t | P |
| --- | --- | --- | --- | --- | --- | --- |
| Spring 2020 | (Intercept) Alb | 559.33 | 102.09 | 24.96 | 5.48 | < 0.01 |
|  | Hai | -357.19 | 78.12 | 42.93 | -4.57 | < 0.01 |
|  | Species richness | -10.49 | 3.77 | 43.69 | -2.78 | 0.01 |
|  | Hai : Species richness | 12.04 | 5.20 | 43.44 | 2.31 | 0.03 |
|  | Soil moisture | -4.70 | 1.87 | 20.31 | -2.52 | 0.02 |
|  | Potential productivity | 108.61 | 436.30 | 44.00 | 0.25 | 0.80 |
|  | LUI | -9.92 | 22.83 | 24.50 | -0.44 | 0.67 |
| Summer 2020 | (Intercept) Alb | -29.84 | 159.34 | 26.19 | -0.19 | 0.85 |
|  | Hai | 97.74 | 92.15 | 43.36 | 1.06 | 0.30 |
|  | Species richness | 1.63 | 5.24 | 40.09 | 0.31 | 0.76 |
|  | Hai : Species richness | 3.33 | 6.46 | 43.77 | 0.52 | 0.61 |
|  | LUI | -29.16 | 31.31 | 23.20 | -0.93 | 0.36 |
|  | Soil moisture | 2.65 | 3.39 | 22.78 | 0.78 | 0.44 |
|  | Potential productivity | 134.65 | 540.30 | 38.94 | 0.25 | 0.81 |
| Spring 2021 | (Intercept) Alb | 185.50 | 60.00 | 20.84 | 3.09 | 0.01 |
|  | Hai | 2.44 | 29.31 | 37.41 | 0.08 | 0.93 |
|  | Species richness | -1.46 | 1.48 | 36.34 | -0.99 | 0.33 |
|  | Hai : Species richness | -1.37 | 1.68 | 33.76 | -0.82 | 0.42 |
|  | Soil moisture | -1.48 | 1.18 | 19.41 | -1.25 | 0.22 |
|  | Potential productivity | 137.00 | 152.70 | 33.73 | 0.90 | 0.38 |
|  | LUI | -16.91 | 8.81 | 18.57 | -1.92 | 0.07 |
| Summer 2021 | (Intercept) Alb | 200.85 | 192.31 | 23.68 | 1.04 | 0.31 |
|  | Hai | 112.77 | 140.40 | 34.52 | 0.80 | 0.43 |
|  | Species richness | 3.95 | 7.46 | 34.14 | 0.53 | 0.60 |
|  | Hai : Species richness | -3.99 | 7.87 | 35.89 | -0.51 | 0.62 |
|  | Soil moisture | -3.75 | 3.20 | 19.75 | -1.17 | 0.26 |
|  | Potential productivity | -666.14 | 718.82 | 36.70 | -0.93 | 0.36 |
|  | LUI | 16.86 | 33.81 | 18.73 | 0.50 | 0.62 |

**Table S17:** Sensitivity analysis of linear model showing the effect of Delta species richness (between reduced land-use + seed addition and reduced land-use subplots) on Delta biomass produced (g m^-2^, between reduced land-use + seed addition and reduced land-use subplots), only for the Schorfheide-Chorin (only new seeds sown), for each year and season.

|  | **Variable** | **Estimate** | **Std. Error** | **t** | **P** |
| --- | --- | --- | --- | --- | --- |
| Spring 2020 | (Intercept) | -55.41 | 20.11 | -2.76 | 0.02 |
|  | Delta species richness | 3.56 | 10.29 | 0.35 | 0.74 |
|  | Delta species richness (quadratic) | 5.11 | 3.27 | 1.56 | 0.15 |
| Summer 2020 | (Intercept) | 8.06 | 27.46 | 0.29 | 0.78 |
|  | Delta species richness | 2.36 | 4.17 | 0.57 | 0.58 |
| Spring 2021 | (Intercept) | -1.04 | 19.20 | -0.05 | 0.96 |
|  | Delta species richness | -1.81 | 4.12 | -0.44 | 0.67 |
| Summer 2021 | (Intercept) | -2.88 | 36.67 | -0.08 | 0.94 |
|  | Delta species richness | 1.76 | 6.44 | 0.27 | 0.79 |

**Table S18:** Sensitivity analysis of linear model showing the effect of Delta species richness (between reduced land-use + seed addition and reduced land-use subplots) on Delta biomass produced (g m^-2^, between reduced land-use + seed addition and reduced land-use subplots), only for the Schwäbische Alb and Hainich-Dün (mixture of resident and new seeds sown), for each year and season.

|  | **Variable** | **Estimate** | **Std. Error** | **t** | **P** |
| --- | --- | --- | --- | --- | --- |
| Spring 2020 | (Intercept) Alb | -7.00 | 12.92 | -0.54 | 0.59 |
|  | Hai | -27.96 | 19.53 | -1.43 | 0.17 |
|  | Delta species richness | -1.96 | 3.34 | -0.59 | 0.56 |
| Summer 2020 | (Intercept) Alb | -12.99 | 15.37 | -0.85 | 0.41 |
|  | fRegionHai | 40.79 | 25.80 | 1.58 | 0.13 |
|  | Delta species richness | -0.89 | 4.28 | -0.21 | 0.84 |
| Spring 2021 | (Intercept) | 2.57 | 5.60 | 0.46 | 0.65 |
|  | Delta species richness | -0.59 | 2.36 | -0.25 | 0.81 |
|  | Delta species richness (quadratic) | -0.26 | 0.32 | -0.82 | 0.42 |
| Summer 2021 | (Intercept) | -31.75 | 18.29 | -1.74 | 0.09 |
|  | Delta species richness | 2.93 | 2.93 | 1.00 | 0.33 |

**Table S19:** Linear model testing in potential productivity between the present land-use treatment covering the reduced gradient in land-use intensity, the reduced land-use treatment and the reduced land-use + seed addition treatment.

| **Variable** | **Estimate** | **Std. Error** | **t** | **P** |
| --- | --- | --- | --- | --- |
| (Intercept) present LU. | 0.024 | 0.003 | 9.07 | < 0.01 |
| Red. LU | -0.003 | 0.004 | -0.92 | 0.36 |
| Red. LU + S | 0.001 | 0.004 | 0.32 | 0.75 |

**Table S20:** Information about environmental variables and calibration data to calibrate biomass estimates accessed via the Biodiversity Exploratories database. All datasets are present in the BExIS database https://www.bexis.uni-jena.de/ (<http://doi.org/10.17616/R32P9Q>). Some of the datasets are already public and some are still under the three years embargo period. Shown here is a description of the datasets provided by the data authors in BExIS metadata. Edited for readability.

| **Dataset ID in BExIS** | **Used for variables** | **Description provided by data authors in BExIS metadata** |
| --- | --- | --- |
| 31180 | Calibration data for calibrating biomass estimations (measured via rising plate-meter) | To create a dataset of a large-scale long-term time series of vegetation composition we conduct an annual monitoring of plant species richness and community composition. In spring of the years 2008-2020, we sampled all species in an area of 4m x 4m and estimated the percentage cover of each species. This area is not disturb by other experiment and is called core area ("Heilige Fläche (HF)" in German).  Biomass was clipped in two subplots. In each subplot we clipped all plants of two 1m x 1m plots at the height of 4cm.  The height was measured as well with a platemeter tool (rising plate meter).  **Authors procedure:** Only Biomass data collected in spring 2021 was used to compute calibration regressions. |
| 24766 | Soil moisture | Climate Data Set - Time Series Web Interface - a new climate data base system, includes all raw values from Advanced environmental monitoring units (AEMUs) + enhanced EMUs (EEMUs) + core EMUs (CEMU) - Automated environmental monitoring by including air pressure, wind, precipitation and radiation at 10 min resolution - aggregated to 1hour + 1day + 1week + 1month + 1year  **Instruments:** ADL-MX Datalogger System; DeltaT ML2X Soil Humidity Probe; MNT FExtension 2010 Soil and Ground Surface Temperature Sensor; MELA KPC1/5-ME & #IAK1.00.F137.520.CS8  Procedure: quality 0 - no quality check  quality 1 - just physical range check  quality 2 - physical range + step range check  quality 3 - physical range + step + empirical check  **Authors procedure:** Daily aggregation was used for soil moisture for the time series 2020-2021. |

**Table S21:** Ratio of mean total cover of sown new species (not present at the site but at the regional species pool) versus unsown species (present at the site) in the reduced land-use + seed addition treatment for each region (Alb: Schwäbische Alb; Sch: Schorfheide-Chorin; Hai: Hainich-Dün) and season.

| **Region** | **Season** | **Ratio of mean total cover of sown/unsown species** |
| --- | --- | --- |
| Alb | Spring | 1.72 |
|  | Summer | 1.81 |
| Hai | Spring | 2.89 |
|  | Summer | 2.58 |
| Sch | Spring | 2.72 |
|  | Summer | 2.47 |
